# UKEV Forum 2024: the UK Society for Extracellular Vesicles Annual Meeting - Abstracts

**DOI:** 10.20517/evcna.2025.100

**Published:** 2025-11-19

**Authors:** Hannah K. Jackson, Naveed Akbar, Nick Peake, Ryan C. Pink, Charlotte Lawson

**Affiliations:** ^1^Children’s Brain Tumour Research Centre, Biodiscovery Institute, Nottingham NG7 2UH, United Kingdom.; ^2^School of Medicine, Biodiscovery Institute, University of Nottingham, Nottingham NG7 2RD, United Kingdom.; ^3^Division of Cardiovascular Medicine, Radcliffe Department of Medicine, University of Oxford, John Radcliffe Hospital, Oxford OX3 9DU, United Kingdom.; ^4^Biomolecular Sciences Research Centre, Sheffield Hallam University, Sheffield S1 1WB, United Kingdom.; ^5^Department of Biological and Medical Sciences, Faculty of Health and Life Sciences, Oxford Brookes University, Oxford OX3 0FL, United Kingdom.; ^6^School of Pharmacy and Biomedical Sciences, University of Central Lancashire, Preston PR1 2HE, United Kingdom.

The UKEV Forum 2024, the annual meeting of the UK Society for Extracellular Vesicles, was held from December 16 to 18, 2024 [Table 1].

**Table 1:** Table of contents

**No.**	**Abstract title**	**Authors**	**Page**
1	Plasma small extracellular vesicles enriched for microglial origin as biomarkers for dementia with Lewy bodies	Fatma Busra Isik	4
2	Extracellular vesicles mediate macrophage metabolic dysfunction in fibrotic and inflammatory lung diseases	Rahul Y. Mahida, Katie L. Spencer, Charlie Mafham, Aaron Scott, Dhruv Parekh, Joy Edwards-Hicks, Mark Lindsay, David R. Thickett	5
3	Pulmonary extracellular vesicles drive alveolar macrophage dysfunction in acute respiratory distress syndrome	Katie L. Spencer, Dhruv Parekh, Mark Lindsay, David R. Thickett, Aaron Scott, Rahul Y. Mahida	6
4	Characterising the contribution of miRNA cargo from syncytiotrophoblast-derived extracellular vesicles to the pathogenesis of pre-eclampsia	Morganne Wilbourne, Alexander Clarke, Shuo Gu, Manu Vatish	6
5	The HSPB1-p62/SQSTM1 functional complex regulates EV-mediated unconventional secretion and transcellular spreading of the HD-associated mutant huntingtin protein	Raffaella Bonavita, Gianluca Scerra, Rosaria Di Martino, Silvia Nuzzo, Elena Polishchuk, Mariagrazia Di Gennaro, Sarah V. Williams, Maria Gabriella Caporaso, Carmen Caiazza, Roman Polishchuk,Massimo D’Agostino, Angeleen Fleming, Maurizio Renna	7
6	Cryo-TEM as a characterisation method for extracellular vesicles in dairy products	Saskia Bakker	8
7	Monitoring the electroactive cargo of extracellular vesicles can differentiate various cancer cell lines	Chloe Miller, Mareike Hermann, David Carter, Nicholas Turner, Priya Samuel, Bhavik Patel	8
8	Do small extracellular vesicles (sEVs) have a role in chronic diabetic foot ulcers?	Lucinda Cruddas, Charlotte Lawson, Markella Ponticos, Janice Tsui	9
9	Interleukin-1β priming induces an anti-inflammatory mesenchymal stem cell phenotype and extracellular vesicle response: implications for NIK-dependent mechanisms and equine musculoskeletal therapies	Emily Clarke, Natasha Poulter, Alison Beckett, Gregory Dykes	9
10	Comparative profiling of mesenchymal stem cell extracellular vesicles for application in regenerative dermatology	Soraya Williams, Emma Buchan, Lee A. Gethings, Rebecca Lees, Pola Goldberg Oppenheimer, Mark P. Lewis, Kate Goldie, Owen G. Davies	10
11	Investigating the abundance of antimicrobial proteins in subtypes of human urinary extracellular vesicles	Tim Williams, Amelia Williams-Walker	11
12	Visualising Parkinson’s disease at the nanoscale	Noelia Pelegrina-Hidalgo, Tilo Kunath, Mathew H. Horrocks	11
13	Unveiling the potential of placental-enriched small extracellular vesicles as modulators of immune homeostasis in spontaneous preterm birth	Himadri Devvanshi, Shinjini Bhatnagar, Garbh-Ini team, Pallavi Kshetrapal, Manu Vatish	12
14	Symptomatic menopausal women not responding to hormone replacement therapy may face increased bone and cardiovascular risk: a pilot study investigating extracellular vesicle protein cargo	Alice Hodge, Emily-Jayne Clarke, Anders Jensen, Olivia Pigden, Paula Briggs, Dharani Hapangama, Mandy Peffers	12
15	Exposure of extracellular vesicles to hyperglycaemic conditions alters surface characteristics and cellular uptake	Charlotte Boyd, Samrein Ahmed, Daniel Kelly, Christine Le Maitre, Nicholas Peake	13
16	Multi-targeted bone regeneration: the role of bone-derived matrix vesicles	Mimma Maggio, Mathieu Y. Brunet, Cansu Gorgun, Rawan Almasri, Lorraine O’Driscoll, David A. Hoey	14
17	Impact of downstream bioprocessing on the identity and immunomodulatory potency of mesenchymal stromal cell-derived extracellular vesicles	Krishalle Richmond, Melanie Moore, Yuan Zhao, Murat Eravci, Min Fang, Lesley Smyth, Samir Shawgy Ayoub, Anna Nowocin	14
18	Optimising immunocapture of extracellular vesicles from cerebrospinal fluid for biomarker discovery in neurodegenerative diseases	Elizabeth R. Dellar, Iolanda Vendrell, Roman Fischer, Alexander G. Thompson	15
19	Maternal extracellular vesicles promote fetal overgrowth in pregnancies complicated by gestational diabetes	Manon D. Owen, Rachel Quilang, Abigail R. Byford, Leander Stewart, Melanie Reay, Naima Endesh, Simon Futers, Neil Turner, Nadira Yuldasheva, Jayne Charnock, Rebecca Spencer, Niamh Forde, Lee Roberts, Beth Holder, Susan Ozanne, Eleanor M. Scott, Karen Forbes	16
20	Small extracellular vesicles released by cardiac fibroblasts from hypertrophic cardiomyopathy patients alter calcium transients and contribute to hypertrophy of human cardiomyocytes	Georgina H. Thompson, Rahul Sanwlani, Elias Sulaiman, Sean Davidson, Konstantinos Savvatis, Aled Clayton, John McVey, Patrizia Camelliti	17
21	Selective attenuation of extracellular vesicle secretion from prostate cancer cells	Jessica Willis, Haiyan An, Demi Pritchyard, Stephen J. Evans, Shareen H. Doak, Laura E. Thomas, Jason P. Webber	17
22	ALK5 inhibitor SD-208 exhibits antifibrotic activity by reverting myofibroblasts to fibroblasts in hypertrophic cardiomyopathy and inhibiting small extracellular vesicle secretion	Rahul Sanwlani, Georgina H. Thompson, Elias Sulaiman, Sean Davidson, Aled Clayton, John McVey, Konstantinos Savvatis, Patrizia Camelliti	18
23	Extracellular vesicles as key cellular messengers: integrating inflammation and tissue repair processes	Ritu Raj, Samuel Charles Hulme, Ivana Milic, Andrew Devitt	19
24	Extracellular vesicles from mesenchymal-derived bone cells regulate osteoclastogenesis	Mimma Maggio, Carolina Martins, Rawan Almasri, Stéphane Petrousek, Mathieu Y. Brunet, Tara Ní Néill, Conor T. Buckley, Lorraine O’Driscoll, David A. Hoey	19
25	Matrix-bound vesicles for regenerative applications in bone	Genevieve Anghileri, Willemijn DeVoogt, Kait Link, Jarod M. Forer, Sanique M. South, Aveen R. Jalal, Cor S. Seinen, Saida Mebarek, Pieter Vader, Ignacio Martin-Fabiani, Nick J. Willett, Owen G. Davies	20
26	Isolation and characterisation of breast cancer patient-derived stromal extracellular vesicles	Emily J. Moss, Mahmoud K. Eldahshoury, Jessica L. Haigh, James R. Boyne	21
27	Intercellular signalling in obesity: adipose-derived EV miRNA communication with skeletal muscle	Joshua Price, Michael Macleod, Thomas Nicholson, Caitlin Ditchfield, Simon Jones	21
28	Extracellular vesicle glycoproteins for early detection of clinically significant prostate cancer	Demi Pritchard, Anastasia Hepburn, Adriana Buskin, Janne Leivo, Haiyan An, Shareen H. Doak, Laura E. Thomas, Rakesh Heer, Rajan Veeratterapillay, Gokul Kanda Swarmy, Jason P. Webber	22
29	Circulating extracellular vesicles in the pregnant ewe and fetus during late gestation	Natalie Jiraskova, Anna L.K. Cochrane, Rachael C. Crew, Youguo Niu, Abigail L. Fowden, Dino A. Guissani, Priya Samuel, Alison J. Forhead	23
30	Does the expression of CD63 in extracellular vesicles present in serum differ between thyroid pathologies?	Hannah Beattie, James England, John Greenman, Victoria Green	23
31	Development of melt electrowritten fibrous scaffolds with controlled release of osteocyte-derived extracellular vesicles for bone repair	Mathieu Y. Brunet, Carolina S. Martins, Rawan Almasri, Luke Madden, Mimma Maggio, Lorraine O’Driscoll, David A. Hoey	24
32	Isolation and characterisation of extracellular vesicles in malaria: implications for pathogenesis and biomarker discovery	Muna Abubaker, Toby Aaron, Joseph Morgan, Sowmya Chinta, Alex Couchman, Berna Sayan, Niroshini Nirmalan, Arijit Mukhopadhyay	25
33	Müller glia-derived EVs promote recovery in retinal ganglion-like cells derived from hESC retinal organoids *in vitro*	Karen Eastlake, William Lamb, Hari Jayaram, Astrid G. Limb	25
34	Impact of drug-to-lipid ratio on microfluidic production of DOX·HCL encapsulating liposomes	Toby M. Hyden-Shepherd, Goran T. Vladisavljevic, Owen G. Davies	26
35	Hijacking breast cancer cell-derived extracellular vesicles for enhanced therapeutic delivery of curcumin	Malika Singh, Ellie Hitchcock, Rebecca Lees, Owen G. Davies	26
36	The Parkinson’s disease-associated protein DJ-1 regulates EV function under oxidative stress by modulating their abundance and protein cargo	Thomas Page, Saskia Bakker, Ivana Milic, Andrew Devitt, Mariaelena Repici	27
37	Aberrant extracellular vesicle signalling by endothelial cells - a potential pathological mechanism in cerebral small vessel disease (SVD)?	Rebecca L. Robertson, Ronja Kremer, Dani Ruiz Gabarre, Garyfallia Gouna, Anna Adamska, Bethany Geary, Lorraine Work, Joanna Wardlaw, Anna Williams	28
38	The impact of hypoxia on macrophage-derived extracellular vesicles in placental implantation studies	Richard Odusanya, Andrew Devitt, Stephane Gross	28
39	Investigating the role of EVs in wound repair: developing corneal and dermal wound healing models	Sasha Kieran, Abigail Adams, James Wolffsohn, Andrew Devitt	29
40	Active extracellular vesicles - exploring the power of EV enzymes in boosting wound healing and macrophage migration	Abigail Elizabeth Adams, Ivana Milic, Andrew Devitt	29
41	Obese adipose-derived extracellular vesicles drive skeletal muscle atrophy: implications for obesity and age-related muscle loss	Michael Macleod, Caitlin Ditchfield, Tom Nicholson, Josh Price, Simon W. Jones	30
42	Proteomic analysis of *ex vivo* glioblastoma tumour-derived extracellular vesicles	Shaun Hanley, Ourania Gkanatsiou, Kesley Attridge, Andrew Devitt, Ivana Milic	31
43	Investigating the role of S100P in placental extracellular vesicle release and maternal immunomodulation	Natasha Louise Baker, Andrew Devitt, Stephane Gross	31
44	Active extracellular vesicles: towards therapeutic use in wound healing & inflammation	Samuel Charles Hulme, Ritu Raj, Ivana Milic, Andrew Devitt	32
45	Determining the effects of electroporation parameters on extracellular vesicle properties	Malika Singh, Ghazaleh Mazaheri-Tehrani, Ignacio Martin-Fabiani, Mark Lewis, Owen G. Davies	32
46	Characterisation of paediatric thymic Tregs and their extracellular vesicles with potential for immunosuppressive therapy in autoimmune disorders	Anaïs Makos, Jordan Bazoer, Henry Barrett, Rafael Guerrero, Dan Hawcutt, Lesley Smyth, Oksana Kehoe	33
47	Characterising the proteomes of *Salmonella* outer membrane vesicles under intestinal conditions	Alastair Scott, Mark Stevens, Garry Blakely, Prerna Vohra	34
48	Measuring surface markers on activated platelet EVs using novel fibre-optic surface plasmon resonance technology	Neline Kriek, Ryan Pink, Jonathan Gibbins	34
49	Maternal obesity programs hearts to release miR-15b-5p in extracellular vesicles after ischemia-reperfusion	Andriana Córdova-Casanova, Lucas C. Pantaleão, Isabella Inzani, Youguo Niu, Dino A. Giussani, Denise S. Fernandez-Twinn, Susan E. Ozanne	35
50	Cyclodextrin’s role in modifying cancer cell extracellular vesicles	Júlia Kvasznicza, Alfrodité Németh, Gréta Bányai, Levente Szőcs, Tamás Garay	35
51	Engineering the next generation of biologically active vascular grafts	Justine Clarke, Mara Boumpouli, Manuel Salmerson-Sanchez, Niall Paterson, Catherine Berry	36
52	Advancing extracellular vesicle analysis: microfluidic approaches for cancer diagnostics	Gréta Lilla Bányai, András Szabó, Anikó Gaál, Csaba Pongor, András Merényi, Tamás Garay	37
53	A characterised immortalised mesenchymal stromal cell (MSC) line to facilitate standardisation of MSC-EV immunomodulatory potency assays	Krishalle Richmond, Melanie Moore, Yuan Zhao, Murat Eravci, Min Fang, Lesley Smyth, Samir Shawgy Ayoub, Anna Nowocin	38
54	Identification of EV biomarkers of senescence and development of sensors to detect them	Benjamin Raven, Caroline Evans, Graham Leggett, Daniel W. Lambert	38
55	How do tumour-derived extracellular vesicles interact with the developing nervous system and alter pain processing in cancer survivors?	Hannah K. Jackson, Anna Grabowska, Victoria James, Federico Dajas-Bailador, Beth Coyle, Gareth Hathway	39
56	Exploring stable sEV miRNA biomarkers for prenatal alcohol exposure using human neural cell models	Toby Aarons, Aadya Lakhani, Amalia Kachramani, Pramitha Sivaprakash, Berna Sayan, Arjit Mukhopadhyay	39
57	Exploring the role of extracellular vesicles in Alzheimer’s disease pathogenesis using Down syndrome *in vitro* models	Ana Muñiz-Garcia, Aoife Murray, Dean Nizetic	40
58	Modulation of extracellular vesicle release from endothelial cells under inflammatory stimulation	Laura Sture, Chong Yun Pang, Neil Sheerin, Simi Ali	41
59	The role of EV-associated miRNAs in medulloblastoma recurrence	Sree V. Dokka, Hannah K. Jackson, Connor T. Levers, Beth Coyle	41
60	Exploring probiotic-derived extracellular vesicles as novel therapeutic agents in the management of inflammatory bowel disease	Robert Matthews, Nouhad El OUazzani, Samir Ayoub, David Guilliano, Nati Garrido Mesa, Lesley Ann Smyth	42
61	Messenger and message: exploring the role of extracellular vesicles in inter-organismal communication during arbuscular mycorrhizal symbiosis in *Medicago*	Serena Qiao, Sam Holland, Alice Boussarogue, Zongyu Gao, Mary Woodward, Ronelle Roth	43
62	Surface glycans of small extracellular vesicles *in vitro* and *in vivo* increase in early- and late-stage colon cancer, providing novel biomarkers for potential blood biopsy	Jamie Cooper, Bethy Airstone, Emanuela Carollo, Susan Ann Brooks, Ryan Pink	43
63	Functional effects of cows’ milk-derived particles on human inflammatory responses *in vitro*	Amy Fenton, Dory Kovacs, Aprill Park, Robert C. Fowkes, Ryan C. Pink, Charlotte Lawson	44
64	Isolation of T cell-derived extracellular vesicles from patients with rheumatoid arthritis	Henry Barrett, Lesley Smyth, Anaїs Makos, Charlotte Hulme, Oksana Kehoe	44
65	Does the secretion of sEVs containing differential microRNA levels by medulloblastoma during disease progression suppress neuroinflammation?	Connor T. Levers, Hannah K. Jackson, Ian D. Kerr, Federico Dajas-Bailador, Beth Coyle	45
66	Bone marrow stromal cell-derived extracellular vesicles for bone regeneration	Evangelia Bochti, Monica P. Tsimbouri, Biswajoy Bagchi, Xin Li, Matthew J. Dalby, Catherine Berry	46
67	Investigating extracellular vesicle heterogeneity during arbuscular mycorrhizal symbiosis in *Medicago* truncatula	Alice Boussarogue, Samuel Holland, Ronelle Roth	46
68	Isolation and characterisation of human amniotic epithelial cell extracellular vesicles for therapeutic repair of human donor hearts and lungs	Nicholas Chilvers, Angel Zhang, Simi Ali, Andrew Fisher, John Dark	47
69	Synovial fluid extracellular vesicles modulate the transcriptome of human articular chondrocytes in osteoarthritis	Caitlin Ditchfield, Joshua Price, Simon Jones	47

## Abstracts Presented at UKEV Forum 2024

The following abstracts were presented at the UKEV Forum 2024 as part of the oral and poster sessions. They highlight the diversity and innovation of ongoing extracellular vesicle (EV) research across basic, translational, and clinical domains.

## Oral presentations

## 1. Plasma small extracellular vesicles enriched for microglial origin as biomarkers for dementia with Lewy bodies

Fatma Busra Isik

Institute of Mental Health, Mental Health and Clinical Neurosciences Academic Unit, University of Nottingham, Nottingham, United Kingdom.

Alzheimer’s disease (AD) and dementia with Lewy bodies (DLB) are the two most common neurodegenerative dementias. They are currently diagnosed by their clinical diagnostic criteria. However, 50% people with DLB may remain misdiagnosed as AD in the UK. Improving clinical diagnosis of DLB is necessary for planning appropriate multidisciplinary care. Hence, there is a need for developing blood-based biomarkers for distinguishing DLB from AD. The heterogeneity of small extracellular vesicles (SEV) poses significant challenges in their isolation, characterisation, and application in biomarker discovery. Overcoming these limitations requires the development of more precise isolation techniques, standardised protocols, as well as a better understanding of the factors contributing to SEV variability. I aimed to isolate and characterise plasma SEV enriched for microglial origin from the extracted SEV from human plasma and to identify differentially expressed genes in plasma SEV, enriched for microglial origin. I aimed to enhance our understanding of molecular mechanisms underlying DLB pathology by completing functional enrichment analysis of the identified differentially expressed genes (DEGs). With the development of an EV enrichment protocol using a Transmembrane Protein 119 (TMEM119) antibody, I successfully achieved up to 20% enrichment for microglial origin from the extracellular vesicles isolated. Furthermore, the analysis of gene expression profiles between individuals with Dementia with Lewy Bodies and Alzheimer’s Disease revealed 28 genes with significant differential expression, 13 of them being downregulated in DLB compared to AD. The ability to analyse SEV-derived RNA represents a substantial methodological improvement, as it mitigates the challenges linked to conventional biomarker identification in biofluids such as plasma.

## 2. Extracellular vesicles mediate macrophage metabolic dysfunction in fibrotic and inflammatory lung diseases

Rahul Y. Mahida^1^, Katie L. Spencer^1^, Charlie Mafham^1^, Aaron Scott^1^, Dhruv Parekh^1^, Joy Edwards-Hicks^2^, Mark Lindsay^3^, David R. Thickett^1^

^1^University of Birmingham, Birmingham, UK. ^2^University of Edinburgh, Edinburgh, UK. ^3^University of Bath, Bath, UK.

Introduction: Alveolar macrophages (AMs) coordinate pulmonary immune responses, and their dysfunction contributes to the pathogenesis of inflammatory {Acute Respiratory Distress Syndrome [ARDS]} and fibrotic {Idiopathic Pulmonary Fibrosis [IPF]} lung disease. We explored the role of extracellular vesicles (EVs) in driving AM dysfunction and metabolic reprogramming.

Methods: Pulmonary EVs were isolated from bronchoalveolar lavage (BAL) fluid of ARDS patients, ventilated controls, and IPF patients. EVs were characterised using nanoparticle tracking analysis (NTA), transmission electron microscopy (TEM), and Exoview. Following ultracentrifugation, pooled EVs were co-cultured with healthy human AMs for functional and metabolic profiling. EV cargo was analysed using transcriptomics, proteomics, and lipidomics.

Results: Treatment of AMs with EVs from ARDS patients impaired efferocytosis (fold change 0.54, *P* = 0.003) compared with control EVs. ARDS EVs also enhanced oxidative phosphorylation and impaired mitophagy. Lipidomic analysis showed elevated levels of lysophosphatidylcholine 18:1 in ARDS EVs, which correlated with increased mitochondrial superoxide release. Transcriptomic profiling revealed enrichment of 8 microRNAs in ARDS EVs, two of which (miR-26b and miR-15a) correlated with 30-day mortality (*P* = 0.04). These microRNAs are associated with mitochondrial dysfunction. Treatment of AMs with miR-26b and miR-15a mimics reproduced the functional and metabolic changes induced by ARDS EVs. In contrast, treatment of human monocyte-derived macrophages (MDMs) with EVs from IPF patients impaired efferocytosis (fold change 0.6, *P* = 0.03) and induced a metabolic shift toward glycolysis. Epithelial-derived EVs were particularly enriched in IPF patients. These EVs showed elevated levels of miR-21 and miR-33a [relative quantification (RQ) 5 *vs.* 20 × 10^4^, *P* = 0.02], both associated with metabolic reprogramming and pro-fibrotic phenotypes.

Conclusions: Targeting EV-associated microRNAs may help restore macrophage homeostasis and offer a novel therapeutic strategy for inflammatory and fibrotic lung diseases.

## 3. Pulmonary extracellular vesicles drive alveolar macrophage dysfunction in acute respiratory distress syndrome

Katie L. Spencer^1^, Dhruv Parekh^1^, Mark Lindsay^2^, David R. Thickett^1^, Aaron Scott^1^, Rahul Y. Mahida^1^

^1^University of Birmingham, Birmingham, UK. ^2^University of Bath, Bath, UK.

Introduction: Acute Respiratory Distress Syndrome (ARDS) is a hyperinflammatory lung disorder that develops secondary to sepsis. We previously found that alveolar macrophages (AMs) from ARDS patients exhibit impaired efferocytosis compared to ventilated sepsis controls (PMID:34112730). This defect is replicated when healthy AMs are treated with bronchoalveolar lavage fluid (BAL) from ARDS patients (PMID:34660643). Impaired AM efferocytosis contributes to alveolar inflammation through the secondary necrosis of apoptotic neutrophils. Experimental lung injury models have implicated extracellular vesicles (EVs) in ARDS pathogenesis. We therefore hypothesised that BAL EVs drive AM dysfunction in ARDS.

Methods and results: EVs were isolated from BAL fluid of ARDS patients and postoperative controls via ultracentrifugation and characterised by nanoparticle tracking analysis (NTA). AMs were isolated from lung tissue resections of non-smoking patients. AMs were treated with BAL EVs for 24 h before efferocytosis assays and Seahorse XF metabolic analysis. ARDS BAL EV treatment impaired AM efferocytosis (fold change 0.541, *P* = 0.0029). ARDS BAL EV-treated AMs showed increased basal oxygen consumption rate (OCR) (*P* = 0.0391), maximal respiration (*P* = 0.0273), and total adenosine triphosphate (ATP) production (*P* = 0.0312). ATP generation increases were driven by enhanced mitochondrial respiration (*P* = 0.0039). Immunocytochemistry suggested that impaired mitophagy underlies the altered mitochondrial activity observed after ARDS EV exposure. Transcriptomic exposure revealed enrichment of 8 microRNAs in ARDS BAL EVs compared to postoperative controls. Among these, BAL EV miR-15a was associated with increased mortality, while both miR-15a and miR-26b were linked to impaired *ex vivo* AM efferocytosis. Transfection of healthy AMs with miR-15a and miR-26b mimics impaired efferocytosis, confirming their functional role.

Conclusions: Targeting the microRNA cargo of EVs may represent a therapeutic strategy to attenuate AM dysfunction and inflammation in ARDS.

## 4. Characterising the contribution of miRNA cargo from syncytiotrophoblast-derived extracellular vesicles to the pathogenesis of pre-eclampsia

Morganne Wilbourne^1^, Alexander Clarke^1^, Shuo Gu^2^, Manu Vatish^1^

^1^University of Oxford, Oxford, UK. ^2^National Cancer Institute (NIH), Bethesda, MD, USA.

Pre-eclampsia (PE) is defined as new-onset hypertension after 20 weeks of gestation, accompanied by end-organ dysfunction (e.g., proteinuria). The placenta releases large quantities of extracellular vesicles (EVs), with levels increasing throughout pregnancy. Previous research has shown alterations in EV size and cargo in gestational disorders, including PE. This project used ncRNA sequencing (ncRNA-seq) to compare microRNA (miRNA) profiles of placental tissue with those of small EVs (sEVs) and medium/large EVs (m/lEVs). Significant differences were observed between placental and EV-associated miRNA profiles. Many miRNAs were underrepresented in EVs relative to their placental expression, while certain key miRNAs were enriched in EVs. Both computationally predicted and experimentally validated targets of these EV-enriched miRNAs were identified. Gene Ontology (GO) analysis showed that targets of EV-enriched miRNAs in normotensive pregnancies were primarily associated with metabolism, respiration, and growth, whereas those in PE were more strongly linked to inflammation and immune responses, consistent with PE as a state of sterile inflammation. Weighted gene coexpression network analysis (WGCNA) identified modules of miRNA/messenger RNA (mRNA) interactions related to PE in sEVs, medium-to-larger EVs (m/lEVs), and placenta tissue. These may represent pathways dysregulated in the disease and could affect maternal cells that internalise syncytiotrophoblast-derived EVs. Future work will assess the effects of EV-enriched miRNAs on recipient cells relevant to PE pathophysiology, including renal cells.

## 5. The HSPB1-p62/SQSTM1 functional complex regulates EV-mediated unconventional secretion and transcellular spreading of the HD-associated mutant huntingtin protein

Raffaella Bonavita^1^, Gianluca Scerra^1^, Rosaria Di Martino^2^, Silvia Nuzzo^3^, Elena Polishchuk^4^, Mariagrazia Di Gennaro^1^, Sarah V. Williams^5^, Maria Gabriella Caporaso^1^, Carmen Caiazza^1^, Roman Polishchuk^4^, Massimo D’Agostino^1^, Angeleen Fleming^5^, Maurizio Renna^1^

^1^University of Naples “Federico II”, Naples, Italy. ^2^Institute of Biochemistry and Cell Biology, National Research Council, Naples, Italy. ^3^IRCCS SYNLAB SDN, Naples, Italy. ^4^Telethon Institute of Genetics and Medicine (TIGEM), Naples, Italy. ^5^Department of Physiology, Development and Neuroscience, University of Cambridge, Cambridge, UK.

Conformational diseases represent a major class of neurological disorders characterised by the aggregation and progressive accumulation of proteins with aberrant conformations. Huntington’s disease (HD) is an autosomal dominant disorder caused by the abnormal expansion of the polyglutamine (polyQ) tract in the huntingtin protein (HTT), which leads to the formation of inclusion bodies in neurons. To date, no effective therapeutic strategy for HD has been established. Recent experimental evidence challenges the conventional view that HD pathogenesis results solely from the intracellular accumulation of mutant protein aggregates. These studies reveal that mutated HTT can undergo transcellular transfer, seeding oligomerisation and aggregation of even the wild-type (WT) protein. Here, we describe a novel functional role of the heat-shock protein beta 1 (HSPB1)-Sequestosome 1 (p62/SQSTM1) complex, which acts as a cargo-loading platform that enables the unconventional secretion of mutant HTT via extracellular vesicles (EVs). HSPB1 preferentially interacts with polyQ-expanded HTT and modulates its oligomerisation and aggregation. Moreover, HSPB1 expression levels correlate with the rate of mutant HTT secretion, which occurs in a multivesicular body (MVB)-dependent manner and is regulated by the phosphatidylinositol 3-kinase (PI3K)/protein kinase B (AKT)/mammalian target of rapamycin (mTOR) signalling pathway. Finally, we show that HTT-containing vesicular structures are biologically active, can be internalised by recipient cells, and thus provide a mechanism for the prion-like spreading properties of mutant HTT.

In addition to investigating the regulation of EV-mediated secretion *in vivo*, we are also assessing whether this process contributes to the turnover of other aggregation-prone, disease-associated proteins.

## 6. Cryo-TEM as a characterisation method for extracellular vesicles in dairy products

Saskia E. Bakker

University of Warwick, Warwick, UK.

Introduction: Extracellular vesicle (EV) enrichment and purification methods often fail to distinguish EVs from other extracellular particles with similar biophysical characteristics. While EV-specific protocols exist, conventional transmission electron microscopy requires dehydration of samples, which alters EV structure and complicates membrane identification. In this study, I investigated the presence of EVs in commercially available dairy products as a model system for examining EVs and non-vesicular extracellular particles (NVEPs) in other biological samples.

Methods and results: Commercially available dairy products were diluted in 0.9% saline. Diluted samples were applied to glow-discharged holey carbon grids and plunge-frozen in liquid ethane. Imaging was performed using a JEOL 2100Plus Transmission Electron Microscope (TEM) with a Gatan OneView camera. Cryo-transmission electron microscopy (cryo-TEM) enabled a clear distinction between EVs and NVEPs, likely casein aggregates, in semi-skimmed, homogenised, pasteurised cow’s milk. Membrane-bound proteins were visible on the vesicle surface, with diameters ranging from 20 nm to 500 nm. Multiple lipid membrane layers were identified in milk fat globules, while NVEPs, putatively casein particles, lacked membrane boundaries. In whipping cream, the proportion of NVEPs and unilamellar EVs was lower compared to milk fat globules, while in yoghurt, the proportion of NVEPs increased and bacterial structures were observed.

Conclusions: This work confirms that cryo-TEM is a desirable method for characterising the size, morphology, and other properties of EVs and NVEPs in purified samples.

## 7. Monitoring the electroactive cargo of extracellular vesicles can differentiate various cancer cell lines

Chloe Miller^1^, Mareike Herrmann^1^, David Carter^2^, Nicholas Turner^3^, Priya Samuel^2^, Bhavik Patel^l^

^1^University of Brighton, Brighton, UK. ^2^Oxford Brookes University, Oxford, UK. ^3^University of Sheffield, Sheffield, UK.

Introduction: Extracellular vesicles (EVs) are pivotal in cell-to-cell communication due to the diverse cargo they carry. EVs are increasingly recognised as important biomarkers for disease identification. However, most current analytical approaches focus primarily on monitoring specific macromolecular targets on the vesicle surface.

Methods and results: Our study focuses on exploring the electroactive components within the cargo of EVs obtained from various cancerous and non-cancerous cell lines using a disk carbon-fibre microelectrode. Distinct variations in oxidizable components were observed when total EV cargo was analysed, with the highest current detected in EVs from MCF7 cells. Furthermore, differences in the types of oxidizable species were identified between EVs from MCF7 and A549 cells. Single-entity measurements showed clear electrochemical spikes corresponding to the detection of oxidizable cargo within EVs from both MCF7 and A549 cells.

Conclusions: These findings highlight the promise of monitoring EV cargo through its electroactive components and suggest new strategies for the specific detection of EVs in the diagnosis and prognosis of various diseases.

## 8. Do small extracellular vesicles (sEVs) have a role in chronic diabetic foot ulcers?

Lucinda Cruddas, Charlotte Lawson, Markella Ponticos, Janice Tsui

University of Central Lancashire, Lancashire, UK.

Introduction: Diabetic foot ulcers develop when wounds fail to progress through the normal physiological stages of healing. They are associated with infection, gangrene, amputation, and high mortality. Small extracellular vesicles (sEVs), which contain proteins, lipids, RNA, and DNA, may contribute to both wound healing and the persistence of chronic pathological wounds.

Methods: *In vitro* analyses were performed on three diabetic and three control human dermal fibroblast cell lines. Each cell line was subjected to combinations of normoxic/hypoxic, normoglycaemic/hyperglycaemic, and inflammatory/non-inflammatory conditions. sEVs were isolated from cell culture media by ultracentrifugation, and their presence, size, and concentration were confirmed using transmission electron microscopy (TEM), nanoparticle tracking analysis (NTA), and Western and dot blot assays.

Results: TEM, NTA, and immunoblotting confirmed the presence of sEVs. Diabetic fibroblasts produced significantly larger sEVs than control fibroblasts under normoglycaemic conditions (*P* = 0.03) but significantly smaller sEVs under hyperglycaemic conditions (*P* = 0.01). In hypoxic conditions, diabetic fibroblasts also produced significantly smaller sEVs compared to controls (*P* = 0.03). No significant size differences were observed between diabetic and control sEVs under inflammatory versus non-inflammatory conditions. Additionally, sEVs from diabetic fibroblasts were significantly smaller in hyperglycaemic conditions compared to normoglycaemic controls (*P* = 0.02). Control cells produced significantly fewer sEVs under hypoxic conditions relative to normoxic conditions (*P* = 0.03).

Conclusions: The concentration, size, packaging, surface protein composition, and release dynamics of sEVs may be influenced by glycaemic state and oxygen availability. Epigenetic changes in diabetic fibroblasts may further affect sEV production, potentially contributing to the pathogenesis of chronic diabetic ulcers.

## 9. Interleukin-1β priming induces an anti-inflammatory mesenchymal stem cell phenotype and extracellular vesicle response: implications for NIK-dependent mechanisms and equine musculoskeletal therapies

Emily Clarke, Natasha Poulter, Alison Beckett, Gregory Dykes

University of Liverpool, Liverpool, UK.

Introduction: Equine mesenchymal stem cells (MSCs) show promise for treating joint disease and tendinopathies, mainly through the paracrine secretion of regenerative factors. Priming MSCs can enhance their therapeutic potential by promoting an anti-inflammatory phenotype and creating a more favourable environment for tissue regeneration. MSC-derived extracellular vesicles (EVs), which mediate cell-cell communication and maintain cellular homeostasis, offer a potential cell-free therapeutic for musculoskeletal diseases. We hypothesised that priming MSCs would induce an anti-inflammatory phenotype reflected in their EV cargo, thereby enhancing their therapeutic value as biologics.

Methods: Equine bone marrow MSCs from three male donors were primed with interleukin-1β. EVs were isolated by ultracentrifugation and characterised using nanoparticle tracking analysis (Nanosight), transmission electron microscopy (TEM), quantitative reverse transcription-polymerase chain reaction (qRT-PCR), and Western Blotting. Protein content was analysed by mass spectrometry and bioinformatic approaches.

Results and discussion: Primed MSCs showed increased expression of immunomodulatory factors, including interleukins, prostaglandin E2, and transforming growth factor-β (TGF-β). A total of 200 proteins were differentially expressed in MSCs and 91 in their EVs. Ingenuity Pathway Analysis identified activation of immune response pathways and cytokine signalling. While EVs lacked immunomodulatory markers detected in MSCs, they contained 73 unique proteins. Among these, nuclear factor-kappa B (NF-κB)-inducing kinase (NIK) was identified as an activated upstream regulator, promoting the secretion of immunosuppressive factors, reducing inflammation, and supporting MSC functional adaptation.

Conclusion: Interleukin-1 beta (IL-1β) priming induces an anti-inflammatory phenotype in MSCs. Although this effect is not directly conserved in EVs, the presence of NIK-associated proteins suggests that primed MSC-EVs act through alternative mechanisms. These findings suggest the potential of primed MSC-EVs as standalone biologics for treating equine musculoskeletal diseases.

## 10. Comparative profiling of mesenchymal stem cell extracellular vesicles for application in regenerative dermatology

Soraya Williams^1^, Emma Buchan^2^, Lee A. Gethings^3^, Rebecca Lees^4^, Pola Goldberg Oppenheimer^2^, Mark P. Lewis^1^, Kate Goldie^5^, Owen G. Davies^1^

^1^Loughborough University, Loughborough, UK. ^2^University of Birmingham, Birmingham, UK. ^3^Waters Corporation, Milford, MA, USA. ^4^NanoFCM Co., Ltd, Nottingham, UK. ^5^European Medical Aesthetics Ltd, Glasgow, UK.

There is growing interest in developing extracellular vesicle (EV)-based regenerative therapies for dermatological applications. We sought to evaluate the influence of tissue origin on the regenerative potential of EVs for dermatology. EVs were isolated by ultracentrifugation from primary adult bone marrow (BM) and perinatal umbilical cord (UB) mesenchymal stem cells (MSCs). MSCs were cultured for 5 days, followed by a 48-h incubation in StemMACS^TM^ xeno-free human media before conditioned media collection. EV preparations were compared with a commercially available EV product marketed for topical regenerative dermatology. Both in-house and commercial preparations contained EVs, although cryo-transmission electron microscopy (Cryo-TEM) confirmed well-defined vesicles only in the in-house preparations, with minimal EV material detected in the commercial product. Particle concentrations measured by nano-flow cytometry (NanoFCM) differed significantly among preparations (UB-MSC EVs: 3.51 × 10^10^ ± 9.09 × 10^8^/mL; BM-MSC EVs: 2.27 × 10^10^ ± 9.98 × 10^8^/mL; commercial: 9.17 × 10^9^ ± 6.61 × 10^8^/mL). Particle-to-protein (PtP) ratios also varied, with UB-MSC EVs showing the highest purity (5.24 × 10^7^ ± 2.42 × 10^6^ particles/µg protein) and the commercial preparation the lowest (7.46 × 10^6^ ± 1.08 × 10^6^ particles/µg protein). NanoFCM further revealed differential expression of biogenesis markers (CD9, CD63, CD81) across EV types. Raman spectroscopy revealed similar overall spectral profiles within the main biological fingerprint region (400-1,800 cm^-1^), with variations in peak intensity. Mass spectrometry, analysed using PANTHER gene ontology and STRING, identified proteins associated with biological pathways relevant to dermatological regeneration, with comparative upregulation observed in BM-MSC EVs. Overall, these preliminary outcomes suggest that MSC-EVs, particularly those derived from BM and UB sources, hold significant promise for regenerative dermatology applications.

## 11. Investigating the abundance of antimicrobial proteins in subtypes of human urinary extracellular vesicles

Tim Williams, Amelia Williams-Walker

Department of Veterinary Medicine, University of Cambridge, Cambridge, UK.

Introduction: Urinary extracellular vesicles (UEVs) are enriched in antimicrobial proteins and exhibit bactericidal activity. This study investigated the proportion of different UEV subtypes (CD9+, CD63+, CD81+) in human urine that contain the antimicrobial proteins myeloperoxidase (MPO) and dermcidin (DCD). Additionally, the abundance of these proteins in UEVs from two patients with recurrent urinary tract infections (rUTI) was evaluated.

Methods and results: Human urine samples were centrifuged and filtered (with a 220 nm filter) prior to UEV capture on human ExoView tetraspanin chips. UEVs were permeabilised and incubated with fluorescently labelled anti-MPO and anti-DCD antibodies, followed by imaging on the ExoView R100 analyser. Data are presented as median [minimum-maximum]. Urine samples from six healthy donors and two rUTI patients were included. MPO was detected in a higher proportion of CD63+ UEVs than CD9+ UEVs {32 [10-59]% *vs.* 11 [3-25]%; *P* = 0.04}, whereas DCD was present in similar proportions of CD9+ and CD63+ UEVs {10 [2-27]% *vs*. 10 [1-20]%, respectively; *P* = 0.84}. CD81+ UEVs were too low in abundance to permit meaningful analysis. The proportion of UEVs containing MPO and DCD in rUTI patients was numerically comparable to that in healthy donors.

Conclusions: Our data suggest that CD63+ UEVs may have greater bactericidal potential than CD9+ UEVs, although further functional studies are needed to confirm this hypothesis. CD63+ UEVs are believed to originate from the proximal nephron, indicating that proximal tubular epithelial cells may contribute to the production of antibacterial UEVs.

## 12. Visualising Parkinson’s disease at the nanoscale

Noelia Pelegrina-Hidalgo^1^, Tilo Kunath^2^, Mathew H. Horrocks^1^

^1^School of Chemistry, University of Edinburgh, Edinburgh, UK. ^2^School of Biology, University of Edinburgh, Edinburgh, UK.

α-synuclein (αSyn) is a protein implicated in the pathology of Parkinson’s disease (PD) and is a major component of Lewy bodies, the hallmark pathological feature of the disorder. Consequently, αSyn aggregation has been a key focus in PD research, providing insights into disease onset and progression. Extracellular vesicles (EVs) are nanoscale vesicles secreted by all cell types, including neurons, and have been shown to contain αSyn in biofluid samples from people with PD. This finding could be a first step towards developing early diagnostic tools for PD. However, the small size of EVs (~100 nm) presents challenges for their characterisation and the detection of αSyn. Traditional microscopy is limited by the diffraction limit of light (~250 nm), requiring alternative techniques for high-resolution imaging. Super-resolution microscopy, with a resolution of ~20 nm, overcomes this limitation, enabling detailed visualisation of EVs.

This project aims to optimise a protocol for observing EV surfaces and imaging their cargo, while exploring the potential of this technique as a PD diagnostic tool before the onset of motor symptoms. Preliminary results indicate that αSyn levels are elevated in EVs from SH-SY5Y cells overexpressing αSyn, but only when the EVs are permeabilised prior to imaging. DNA-PAINT super-resolution imaging of these samples revealed structural details that cannot be resolved with traditional microscopy. These findings mark an initial advancement in detecting αSyn aggregates in EVs using super-resolution techniques. Future research will focus on analysing EVs derived from the biofluids of individuals living with Parkinson’s disease.

## Poster presentations

## 13. Unveiling the potential of placental-enriched small extracellular vesicles as modulators of immune homeostasis in spontaneous preterm birth

Himadri Devvanshi^1^, Shinjini Bhatnagar^1^, Garbh-Ini team^1^, Pallavi Kshetrapal^1^, Manu Vatish^2^

^1^Department of Maternal Child Health, Translational Health Science and Technology Institute, India. ^2^Nuffield Department of Women’s & Reproductive Health, University of Oxford, Oxford, UK.

Introduction: A stringent balance between immune tolerance and inflammation is essential for a successful pregnancy. During the second trimester (a critical period of fetal development), immune-suppressive cells including regulatory, exhausted, and senescent T cells are augmented. Disruption of immunological homeostasis increases the risk of preterm birth, a common pregnancy complication associated with significant mortality and morbidity. Small extracellular vesicles (sEVs) secreted by the syncytiotrophoblast (STB) layer of the placenta enter maternal circulation, mediating the exchange of biomolecular signals during pregnancy. STB-derived sEVs (30-150 nm) have been implicated in the pathophysiology of many pregnancy complications. In the present study, we investigated the immunomodulatory role of circulating STB sEVs in term (TB) and spontaneous preterm pregnancies (sPTB).

Methods and results: STB sEVs were isolated from maternal plasma of TB and sPTB cases (second trimester) using an antibody against Placental Alkaline Phosphatase - a known marker of placental origin. STB sEVs were co-cultured with peripheral blood mononuclear cells (PBMCs) from healthy donors. T cell activation and suppression were measured by flow cytometry, and these cellular signatures were validated at both the transcript and effector cytokine levels. We observed that STB sEVs from sPTB enhanced pro-inflammatory responses in PBMCs by activating cytotoxic T cells. Regulatory T cells and their molecular signatures were suppressed in sPTB compared to TB (*P* ≤ 0.05).

Conclusions: Placental factors delivered via STB sEVs can disrupt maternal immune homeostasis, thus promoting a pro-inflammatory environment in sPTB.

## 14. Symptomatic menopausal women not responding to hormone replacement therapy may face increased bone and cardiovascular risk: a pilot study investigating extracellular vesicle protein cargo

Alice Hodge^1^, Emily-Jayne Clarke^1^, Anders Jensen^1^, Olivia Pigden^1^, Paula Briggs^2^, Dharani Hapangama^1^, Mandy Peffers^1^

^1^University of Liverpool, Liverpool, UK. ^2^Liverpool Women’s NHS Foundation Trust, Liverpool, UK.

Introduction: Variability in hormone replacement therapy (HRT) efficacy among menopausal women necessitates early identification of responders to optimise clinical outcomes and resource use. Extracellular vesicles (EVs), intercellular messengers released in response to physiological triggers, including hormones, may reflect an individual’s response to HRT. This study is the first to analyse plasma EV proteomes in menopausal women, exploring mechanisms underlying HRT non-responsiveness and their potential as biomarkers.

Methods: In a cross-sectional study (n = 12), participants were grouped by menopausal symptom profiles into “No HRT”, “Implants”, “Standard HRT responders” (SHRT.R), and “Non-responders” (SHRT.NR). EVs were isolated by differential ultracentrifugation and characterised using nanoparticle tracking analysis, transmission electron microscopy, and western blotting, followed by proteomic analysis via mass spectrometry and bioinformatics.

Results: EV characterisation showed a heterogeneous population with sizes ranging from 146.5 nm to 223.5 nm and concentrations of 3.63 × 10^8^ to 5.95 × 10^9^ particles/mL. EV morphology and surface markers (ALIX, CD9) were verified. Among 333 identified proteins, 67 were differentially abundant across groups. SHRT-NR displayed a unique proteome with significant pathway differences. Transforming growth factor-β (TGF-β) was downregulated in SHRT-NR compared to No HRT (*P* = 0.036, z = 1.114) and Implant (*P* = 0.004, z = -2.172), and IL-4 was downregulated in SHRT-NR versus No HRT (*P* = 0.016, z = 0.447) and SHRT.R (*P* = 0.033, z = -0.343). Production (*P* = 0.032, z = 0.830) and synthesis (*P* = 0.048, z = 0.916) of reactive oxygen species, as well as necrosis (*P* = 0.045, z = 1.525), were upregulated in SHRT-NR. Keratin 1 (KRT1), Immunoglobulin Kappa Variable 1-27 (IGKV1-27), and Adenosine deaminase RNA-specific B1 (ADARB1) emerged as potential biomarkers for SHRT-NR.

Conclusion: SHRT-NRs may experience reduced protective effects of HRT on bone and cardiovascular health. Identification of specific protein markers could improve the prediction of individual HRT responsiveness.

## 15. Exposure of extracellular vesicles to hyperglycaemic conditions alters surface characteristics and cellular uptake

Charlotte Boyd^1^, Samrein Ahmed^1^, Daniel Kelly^1,2^, Christine Le Maitre^2^, Nicholas Peake^1^

^1^Sheffield Hallam University, Sheffield, UK. ^2^University of Sheffield, Sheffield, UK.

Introduction: Individuals with type 2 diabetes are 27% more likely to develop colorectal cancer (CRC), and they experience lower survival rates and poorer treatment responses. The underlying mechanisms remain unclear. Extracellular vesicle (EV) uptake is crucial for cell-cell communication, and EVs are involved in the pathogenesis of both diabetes and cancer. However, their role in linking these two diseases has not yet been explored. This study examined the effects of hyperglycaemic conditions on EV surface characteristics and cellular uptake.

Methods: EVs were isolated from SW480 CRC cells grown in CellLine AD-1000 bioreactors using size exclusion chromatography and characterised by dissociation-enhanced lanthanide fluoroimmunoassay (DELFIA) enzyme-linked immunosorbent assay (ELISA) , bicinchoninic acid (BCA) assay, and size profiling. EVs were fluorescently labelled with MemGlow^TM^ 700 and treated with or without 25 mM glucose overnight, and/or with 1mM ALT-711 for 1 h. They were then added to SW480 cells cultured in Plasmax media, which mimics physiological metabolic conditions. After 24 h, EV uptake was assessed by flow cytometry, and zeta potential was measured using ZetaView (Particle Metrix).

Results: Glucose-treated EVs showed increased surface expression of advanced glycation end products (AGEs), which decreased over time after treatment. The uptake of glucose-treated EVs by cells was reduced, and these EVs also displayed a lower zeta potential. Treatment with the AGE breaker alagebrium chloride (ALT711) further reduced EV uptake by cells.

Conclusions: These findings suggest that glucose treatment alters EV surface chemistry, reducing their cellular uptake. This mechanism may contribute to the link between hyperglycaemia and CRC outcomes.

## 16. Multi-targeted bone regeneration: the role of bone-derived matrix vesicles

Mimma Maggio^1^, Mathieu Y. Brunet^1^, Cansu Gorgun^2^, Rawan Almasri^1^, Lorraine O’Driscoll^1^, David A. Hoey^1,2,3^

^1^Trinity College Dublin, Dublin, Republic of Ireland. ^2^Royal College of Surgeons in Ireland (RCSI), Dublin, Republic of Ireland. ^3^Advanced Materials and Bioengineering Research Centre (AMBER), Dublin, Republic of Ireland.

Matrix vesicles (MVs) are membrane-bound nanoparticles secreted by mature bone cells within the extracellular matrix (ECM). They are essential for biomineralisation, play a critical role in ECM-derived scaffolds for tissue regeneration, and are likely present in bone grafts, which remain the gold standard for bone repair. Despite being discovered in the 1960s, the precise mechanisms, multimodal functions, and classification of MVs as an extracellular vesicle subpopulation remain underexplored. This study aimed to develop a reliable method for isolating MVs directly from animal tissues and to assess their pro-regenerative potential in bone repair.

MVs were isolated from the long bones and kidneys of 5-month-old C57BL/6J mice through tissue digestion, filtration (100 µm), and differential ultracentrifugation (TH660 swinging rotor, 110,000 g, 90 min, 4 °C). Characterisation included nanoparticle tracking analysis (NTA) and alkaline phosphatase (ALP) activity assays. The effects of MV on mineralisation and osteoclastogenesis were assessed by administering graded doses to SaOS-2 osteoblastic cells and human CD14+ monocytes under osteogenic and osteoclastogenic conditions, respectively. NTA showed that long bone-derived MVs (lb-MVs) were significantly smaller than kidney-derived MVs (*P* < 0.0001), though yields were comparable. Both MV types expressed functional ALP, confirming mineralising ability, with lb-MVs showing higher ALP activity (*P* < 0.0001). Moreover, lb-MVs exhibited both anabolic and anti-catabolic effects by promoting mineralisation and inhibiting osteoclastogenesis in a dose-dependent manner.

This study successfully isolated MVs from mouse tissues, providing a relevant model to study their role in bone biology. Notably, lb-MVs exhibited both anabolic and anti-catabolic effects, suggesting their potential applications in bone repair.

## 17. Impact of downstream bioprocessing on the identity and immunomodulatory potency of mesenchymal stromal cell-derived extracellular vesicles

Krishalle Richmond^1,2^, Melanie Moore^1^, Yuan Zhao^1^, Murat Eravci^1^, Min Fang^1^, Lesley Smyth^3^, Samir Shawgy Ayoub^2^, Anna Nowocin^1^

^1^Medicines and Healthcare products Regulatory Agency (MHRA), London, UK. ^2^University of East London, London, UK. ^3^University of West London, London, UK

Introduction: Mesenchymal stromal cells (MSCs) modulate immune cell activity via their secretome, which includes extracellular vesicles (MSC-EVs). Efforts to elucidate the mechanisms of MSC-EV immunomodulation have been complicated by their heterogeneity, which depends on both the cell source and the methods used for EV bioprocessing. This study investigates the impact of EV isolation methods on MSC-EV phenotype and immunomodulatory function, with the aim of identifying critical attributes that enhance reproducibility in therapeutic investigations.

Methods and results: An immortalised MSC (ImmMSC) line, demonstrating phenotypic and immunomodulatory properties similar to those of primary human bone marrow-derived MSCs, was established to standardise upstream EV bioprocessing. EVs were isolated from conditioned media (CM) collected from ImmMSCs using differential ultracentrifugation (DUC), ExoQuick-TC^TM^ (ExoQ) precipitation, size exclusion chromatography (SEC), or tangential flow filtration (TFF). Nanoparticle tracking analysis, bicinchoninic acid (BCA), and multiplex Meso Scale Discovery platform (U-plex MSD) assays showed that, among the tested methods, SEC and ExoQ produced EV-enriched fractions with higher nanoparticle yields and greater expression of EV markers, with SEC providing the highest purity. The immunomodulatory capacity of the secretome and its individual components, MSC-EV and non-vesicular extracellular particles (NVEP), was evaluated by measuring T cell proliferation in a Mixed Lymphocyte Reaction (MLR) assay. Both MSC-EVs and, surprisingly, some NVEP fractions obtained from ExoQ, SEC, and TFF suppressed T cell proliferation in a dose-dependent manner. Conversely, MSC-CM appeared to promote T cell proliferation.

Conclusion: EV isolation methods can affect the identity, purity, and potency of the resulting EV products. Therefore, the choice of isolation method should be carefully matched to the intended application, particularly when assessing EV bioactivity.

## 18. Optimising immunocapture of extracellular vesicles from cerebrospinal fluid for biomarker discovery in neurodegenerative diseases

Elizabeth R. Dellar^1^, Iolanda Vendrell^2,3^, Roman Fischer^2,3^, Alexander G. Thompson^1^

^1^Nuffield Department of Clinical Neurosciences, University of Oxford, Oxford, UK. ^2^Target Discovery Institute, Centre for Medicines Discovery, Nuffield Department of Medicine, University of Oxford, Oxford, UK. ^3^Chinese Academy of Medical Sciences Oxford Institute, Nuffield Department of Medicine, University of Oxford, Oxford, UK.

Introduction: Central nervous system (CNS) cells secrete extracellular vesicles (EVs) into cerebrospinal fluid (CSF), making them a promising source of biomarkers for neurodegenerative diseases. Conventional purification methods such as ultracentrifugation and size exclusion chromatography (SEC) require large sample volumes. Immunocapture offers a potential alternative by reducing sample volume requirements and enabling the identification of cellular origin. This study aimed to optimise immunocapture and proteomic profiling of CNS-EVs from CSF for biomarker discovery.

Methods: EVs were purified from 200-1,000 µL of CSF using M-270 Epoxy Dynabeads conjugated with a cocktail of tetraspanin antibodies, or by SEC. Captured EVs were lysed with 5% sodium dodecyl sulfate (SDS) and digested using Lys-C/trypsin with suspension trapping. Proteomic analysis was performed by liquid chromatography-tandem mass spectrometry with library-free data-independent acquisition.

Results: Immunocapture was effective from CSF volumes as small as 200 µL, consistently detecting EV markers [CD9, CD81, TSG101, Syntenin-1, ALIX, and flotillin-1 (FLOT1)] while reducing contamination by non-vesicular proteins (Apolipoprotein B, LGALS3BP) compared to SEC. Proteomic depth reached 811 ± 14 proteins at 200 µL, increasing to 1,285 ± 224 and 1,266 ± 18 for 500 µL and 1,000 µL, respectively, compared with 812 ± 66 for SEC (500 µL). Identified proteins were enriched for choroid plexus and astrocytic markers, but not neuronal markers. Precursor peptide mapping of proposed neuronal EV capture targets revealed some cytoplasmic-localised peptides, indicating the presence of membrane-bound proteoforms.

Conclusions: This study demonstrates that immunocapture enables robust purification of EVs from small CSF volumes using generic markers. This approach also provides a platform for testing alternative capture targets specific to neuronal, microglial, and astroglial EVs.

## 19. Maternal extracellular vesicles promote fetal overgrowth in pregnancies complicated by gestational diabetes

Manon D. Owen^1^, Rachel Quilang^1^, Abigail R. Byford^1^, Leander Stewart^1^, Melanie Reay^1^, Naima Endesh^1^, Simon Futers^1^, Neil Turner^1^, Nadira Yuldasheva^1^, Jayne Charnock^2^, Rebecca Spencer^1^, Niamh Forde^1^, Lee Roberts^1^, Beth Holder^3^, Susan Ozanne^4^, Eleanor M Scott^1^, Karen Forbes^1^

^1^University of Leeds, Leeds, UK. ^2^Edge Hill University, Ormskirk, UK. ^3^Imperial College London, London, UK. ^4^University of Cambridge, Cambridge, UK.

Introduction: Gestational Diabetes Mellitus (GDM) is frequently associated with large-for-gestational-age (LGA) infants, who have increased cardiometabolic risk compared to appropriate-for-gestational-age (AGA) infants. The underlying mechanisms remain poorly understood. We hypothesised that extracellular vesicles (EVs) and their microRNA (miRNA) cargo influence fetal growth in GDM pregnancies.

Methods and results: EVs were isolated from maternal serum collected from non-GDM pregnancies delivering AGA infants, or from GDM pregnancies resulting in either AGA or LGA infants. EVs were characterised according to Minimal Information for Studies of Extracellular Vesicles (MISEV) guidelines, and EV-associated miRNAs were profiled using quantitative real-time polymerase chain reaction (QPCR) arrays. EVs, vehicle [Performance Based Standards (PBS)], or specific miRNA mimics were delivered to healthy pregnant C57BL6/J mice via tail vein injection. At E18.5, dams were euthanised and fetal and placental weights recorded. Placental morphology was examined by immunohistochemistry, and metabolomics was performed using liquid chromatography-mass spectrometry (LC-MS) . Human placental explants from uncomplicated pregnancies were transfected with miR-375-3p mimics, followed by proteomics tandem mass tag (TMT)-based proteomics and functional enrichment analysis. Delivery of EVs from GDM-LGA pregnancies to the circulation of pregnant mice increased fetal weight. Several miRNAs were altered in GDM-LGA EVs, including miR-375-3p. Similar to GDM-LGA EVs, delivery of miR-375-3p mimics increased both fetal and placental weight compared to controls. miR-375-3p also enlarged the total placental surface area at E18.5 and altered amino acid and lipid metabolism. In human placental explants, miR-375-3p overexpression modified the placental proteome, with differentially expressed proteins mapping to pathways involved in placental growth and metabolism.

Conclusion: Our findings suggest that in GDM pregnancies, maternal EVs deliver miR-375-3p to the placenta, where it promotes fetal overgrowth by altering placental growth and function.

## 20. Small extracellular vesicles released by cardiac fibroblasts from hypertrophic cardiomyopathy patients alter calcium transients and contribute to hypertrophy of human cardiomyocytes

Georgina H. Thompson^1^, Rahul Sanwlani^1^, Elias Sulaiman^2^, Sean Davidson^2^, Konstantinos Savvatis^3^, Aled Clayton^4^, John McVey^1^, Patrizia Camelliti^1^

^1^School of Biosciences and Medicine, University of Surrey, Guildford, UK. ^2^The Hatter Cardiovascular Institute, University College London, London, UK. ^3^Inherited Cardiovascular Diseases Unit, Barts Heart Centre, London, UK. ^4^Division of Cancer & Genetics, School of Medicine, Cardiff University, Cardiff, UK.

Cardiac fibroblasts (CFs) are abundant in the heart and play a major role in cardiac homeostasis and disease. However, the contribution of CF-derived small extracellular vesicles (sEV) to cardiac disease is not well defined. We investigated whether sEVs released from CFs of hypertrophic cardiomyopathy (HCM) patients (HCM-CFs) influence calcium handling and hypertrophy in cardiomyocytes (CMs). CMs were cultured with 0-50 μg/mL HCM-CF sEV for 48 h. Calcium (Ca^2+^) transients were measured by optical mapping with Fluo-4 dye. Everse transcription real-time quantitative polymerase chain reaction (RT-qPCR) was performed to assess expression of Ca^2+^ handling and hypertrophy-related genes. Cell size was measured by immunohistochemical staining for sarcomeric α-actinin. Data were normalised to the 0 μg/mL (no sEV) condition, and percentage changes were calculated. Treatment with 50 μg/mL HCM-CF sEVs significantly shortened Ca^2+^ transient time to peak and time to 50% decay (*P* < 0.05, n = 8). Expression of genes encoding Ca^2+^ handling proteins [ryanodine receptor 2 (RYR2) and Inositol 1,4,5-Trisphosphate Receptor (ITP3R)] and hypertrophy-related genes [atrial natriuretic peptide (ANP)/brain natriuretic peptide (BNP)/α-Myosin Heavy Chain (MYH6)] was significantly increased in CMs treated with 50 μg/mL sEV (*P* < 0.05, n = 6). CM spontaneous beating frequency rose in a dose-dependent manner, with increases of 40% at 10 μg/mL and up to 90% at 50 μg/mL (*P* < 0.001, n = 14-19). CM size increased by 40% following exposure to 20-50 μg/mL HCM-CF sEVs (*P* < 0.001, n = 18). HCM-CF-derived sEVs significantly decrease Ca^2+^ transient duration by upregulating RyR2 and ITP3R. Moreover, they promote hypertrophy in CMs, as demonstrated by increased hypertrophy gene expression, cell enlargement, and elevated spontaneous beating frequency.

## 21. Selective attenuation of extracellular vesicle secretion from prostate cancer cells

Jessica Willis, Haiyan An, Demi Pritchard, Stephen J. Evans, Shareen H. Doak, Laura E. Thomas, Jason P. Webber

Swansea University, Swansea, UK.

Introduction: Each year, more than 52,000 patients are diagnosed with prostate cancer (PCa) in the UK. We previously highlighted a role for specific extracellular vesicle (EV) subsets in driving PCa progression. We hypothesise that therapeutically targeting specific EV subsets has the potential to attenuate PCa progression while limiting the off-target effects associated with broad inhibition of EV secretion.

Methods: The effects of five commercially available drugs/compounds (Y27632, GW4869, Imipramine, Manumycin A, and Calpeptin) on EV secretion from PCa cell lines (LNCAP, DU145, and PC3) and non-cancerous cells (PNT2) were assessed by nanoparticle tracking analysis and immunofluorescence-based detection. Everse transcription real-time quantitative polymerase chain reaction (RT-qPCR) was performed to examine the effects of drug treatment on key regulators of EV biogenesis. The impact of drug treatment on EV phenotype was further explored using immunoaffinity-based approaches.

Results: Drug/compound effects on EV secretion were highly cell type-dependent. Calpeptin appeared to show the greatest specificity in attenuating EV secretion from PCa cell lines, with minimal effect on non-cancerous PNT2 cells. The effects of Calpeptin were therefore compared with those of GW4869, a well-established inhibitor of EV secretion. Treatment with Calpeptin or GW4869 resulted in attenuated messenger RNA (mRNA) expression of several EV biogenesis regulators, such as synaptosome-associated protein of 23 kDa (SNAP23) and secretory carrier membrane protein 3 (SCAMP3). Additionally, drug treatment altered EV phenotype.

Conclusion: These findings highlight the potential of certain compounds to attenuate EV secretion. In particular, Calpeptin appeared to reduce both the number and alter the phenotype of EVs secreted from PCa cell lines, but not from non-cancerous cells.

## 22. ALK5 inhibitor SD-208 exhibits antifibrotic activity by reverting myofibroblasts to fibroblasts in hypertrophic cardiomyopathy and inhibiting small extracellular vesicle secretion

Rahul Sanwlani^1^, Georgina H. Thompson^1^, Elias Sulaiman^2^, Sean Davidson^2^, Aled Clayton^3^, John McVey^1^, Konstantinos Savvatis^4^, Patrizia Camelliti^1^

^1^Faculty of Health and Medical Sciences, School of Biosciences and Medicine, University of Surrey, Guildford, UK. ^2^The Hatter Cardiovascular Institute, University College London, London, UK. ^3^Division of Cancer & Genetics, School of Medicine, Cardiff University, Cardiff, UK. ^4^Inherited Cardiovascular Diseases Unit, Barts Heart Centre, London, UK.

Hypertrophic cardiomyopathy (HCM) is the most prevalent genetic cardiomyopathy. Previously, we reported that paracrine signalling from myofibroblasts regulates the electrophysiology of human induced pluripotent stem cell (hiPSC)-derived cardiomyocytes. Increasing evidence highlights extracellular vesicles (EVs) as important mediators of pathophysiology. To investigate the role of cardiac fibroblasts (CFs) and their EVs in regulating cardiomyocyte function, this study assessed the effects of the ALK5 inhibitor SD-208 (2,4-disubstituted pteridine) on HCM CFs.

CFs were isolated from HCM patient biopsies using tissue explants. SD-208 treatment did not significantly affect the viability or metabolic potential of HCM CFs. However, molecular analysis suggested significant reductions in messenger RNA (mRNA) levels of activation markers [α-smooth muscle actin (ACTA2), periostin (POSTN), fibroblast activation protein (FAP), collagen type 1 alpha 1 gene (COL1A1), interleukin-6 (IL-6), and interleukin-11 (IL-11); *P* < 0.0001]. Flow cytometry showed a significant decrease in alpha-smooth muscle actin (α-SMA)-positive cells (*P* = 0.0043), and total collagen secretion was reduced (*P* = 0.00062). CF EVs, collected by size exclusion chromatography, were within the small EV size range and enriched for EV marker proteins. Notably, SD-208 treatment led to a 6.5-fold reduction in EV secretion by HCM CFs (*P* < 0.0001). Similarly, SD-208 did not affect the human embryonic kidney 293 (HEK-293) cell viability or metabolism, but induced a dose-dependent decrease in EV yield (*P* < 0.01).

Overall, these findings demonstrate the antifibrotic activity of SD-208. By reverting myofibroblasts to fibroblasts, SD-208 provides a valuable tool to study the role of CF EVs in cardiomyocyte regulation. Moreover, this study highlights the potential of SD-208 as a regulator of EV biogenesis and secretion.

## 23. Extracellular vesicles as key cellular messengers: integrating inflammation and tissue repair processes

Ritu Raj^1,2^, Samuel Charles Hulme^2^, Ivana Milic^1,2^, Andrew Devitt^1,2^

^1^Aston Institute for Membrane Excellence, Birmingham, UK. ^2^School of Biosciences, Aston University, Birmingham, UK.

Introduction: Extracellular vesicles (EVs) play a significant role in inflammation by mediating cellular communication and immune regulation. They transport bioactive molecules that can either drive or resolve inflammation, modulate immune responses, and show promise as therapeutic agents. Building on more than a decade of research from the Devitt lab, this study aims to engineer therapeutic EVs with targeted immunomodulatory properties to resolve inflammation and support tissue regeneration.

Methods: Human embryonic kidney (HEK-293-F) cells were genetically modified to express immunomodulatory enzymes (e.g., Lipoxygenases), with expression confirmed by immunoblotting. After EV isolation, characterisation was performed using an array of bioanalytical and biophysical techniques. Their immunomodulatory potential was evaluated through *in vitro* assays.

Results: The modified 293-F cells expressed pro-resolving mediators of inflammation, including lipoxygenase enzymes that regulate inflammatory responses. Characterisation of EVs revealed the presence of surface biomarkers and pro-resolving cargo. EVs secreted by the modified cells transferred active biomolecular components to recipient cells while retaining their metabolic activity.

Conclusion: EVs represent a powerful tool for modulating immune responses, uniquely capable of both initiating and resolving inflammation. This study underscores the potential of EVs as active metabolic regulators that can promote tissue repair and regeneration. As research progresses, EV-based therapies may provide transformative treatment options for inflammatory diseases and chronic wound healing, offering new avenues for targeted and effective interventions in regenerative medicine.

## 24. Extracellular vesicles from mesenchymal-derived bone cells regulate osteoclastogenesis

Mimma Maggio^1^, Carolina Martins^1^, Rawan Almasri^1^, Stéphane Petrousek^1,2^, Mathieu Y. Brunet^1^, Tara Ní Néill^1,2^, Conor T. Buckley^1,2,3^, Lorraine O’Driscoll^1^, David A. Hoey^1,2,3^

^1^Trinity College Dublin, Dublin, Republic of Ireland. ^2^Advanced Materials and Bioengineering Research Centre (AMBER), Dublin, Republic of Ireland. ^3^Royal College of Surgeons in Ireland (RCSI), Dublin, Republic of Ireland.

Introduction: Osteoporosis results from an imbalance in bone remodelling, leading to an increased risk of fractures. Osteocytes regulate osteoclastogenesis through paracrine factors influenced by mechanical loading. Extracellular vesicles (EVs) have recently emerged as key physiological mediators; however, their role in osteoclastogenesis remains unclear. This study investigated the impact of mesenchymal-derived bone cells and their EVs on osteoclastogenesis, focusing on lineage commitment and the influence of mechanical stimulation.

Methods: Human marrow stromal/stem cells (MSCs), human osteoblasts (OBs), and MLO-Y4 osteocyte-like cells (OCYs) were subjected to oscillatory fluid shear (OFS). EVs from static (EV-S) and mechanically stimulated (EV-F) cells were isolated from conditioned media (CM) via ultracentrifugation (70Ti fixed-angle rotor, 110,000 g, 75 min) and characterised using nanoparticle tracking analysis (NTA), transmission electron microscopy (TEM), and flow cytometry. Osteoclast differentiation of human CD14+ monocytes was assessed following treatment with CM or EVs. EV-derived microRNA was sequenced, and selected miRNAs were transfected into monocytes.

Results: CM from MSCs under both static (CM-S) and flow (CM-F) conditions inhibited osteoclastogenesis. While the static OB secretome had no effect, OB-CM-F significantly reduced osteoclast differentiation. Both OCY-CM-S and OCY-CM-F inhibited osteoclastogenesis, with stronger inhibition observed under mechanical stimulation. Following EV isolation, MSC-EVs inhibited osteoclastogenesis independent of the mechanical environment. OB-EVs also inhibited osteoclastogenesis, with stronger effects from EV-F. OCY-EVs exerted potent inhibitory effects, particularly following mechanical stimulation of the parental cells. miR-150-5p, identified in OCY-EVs, was transfected into monocytes and demonstrated a dose-dependent decrease in osteoclastogenesis.

Conclusion: These results highlight osteocytes as key regulators of osteoclastogenesis through their EVs. MSC- and OB-derived secretomes also inhibited osteoclast differentiation to varying degrees, with effects mediated by EVs and influenced by mechanical conditions. This study advances understanding of EV-mediated regulation of osteoclastogenesis and underscores their potential as cell-free therapeutic strategies for osteoporosis.

## 25. Matrix-bound vesicles for regenerative applications in bone

Genevieve Anghileri^1^, Willemijn DeVoogt^2^, Kait Link^3^, Jarod M. Forer^3^, Sanique M. South^3^, Aveen R. Jalal^1^, Cor S. Seinen^2^, Saida Mebarek^4^, Pieter Vader^2^, Ignacio Martin-Fabiani^1^, Nick J. Willett^3^, Owen G. Davies^1^

^1^Loughborough University, Loughborough, UK. ^2^UMC Utrecht, Utrecht, Netherlands. ^3^Phil and Penny Knight Campus for Accelerating Scientific Impact, University of Oregon, Eugene, OR, USA. ^4^Claude Bernard University Lyon 1, Lyon, France.

Introduction: Matrix-bound vesicles (MBVs) function as nucleation sites for mineral formation in developing tissues such as bone. However, therapeutic studies have largely focused on media vesicles (MVs) isolated from suspension cultures. Consequently, the therapeutic potential of MBVs remains largely undefined. This study sought to characterise MBVs and evaluate their therapeutic potential using *in vitro* and *in vivo* models.

Methods: MBVs and MVs were isolated from primary human bone marrow stromal/stem cells (MSCs) cultured under osteogenic conditions and characterised in accordance with Minimal Information for Studies of Extracellular Vesicles (MISEV) guidelines. Mineralisation capacity and composition were assessed using phosphate hydrolysis assays, Fourier transform infrared spectroscopy (FTIR), and transmission electron microscopy (TEM). Binding to collagen and bovine bone hydrogel (dECM) scaffolds was compared to identify suitable delivery systems. *In vitro* and *in vivo* models were employed to quantify extracellular vesicle (EV) clearance [1,1’-dioctadecyl-3,3,3’,3’ tetramethylindotricarbocyanine iodide (DiR) labelled] and mineralisation.

Results: Significantly higher numbers of MBVs were released from the extracellular matrix compared to MVs recovered from conditioned medium. Phosphate-hydrolysing enzymes and channel proteins essential for mineralisation were elevated in MBVs, and their localisation was confirmed by super-resolution microscopy. MBVs rapidly hydrolysed polyphosphate sources and enhanced mineralisation in MSC cultures. They were also enriched in electron-dense material identified as hydroxyapatite. Both *in vitro* and *in vivo* analyses demonstrated that MBVs exhibited significantly greater binding affinity for collagen and dECM scaffolds, whereas MVs showed minimal binding. DiR-labelled MBVs remained detectable on collagen sponges implanted subcutaneously for up to 4 weeks.

Conclusions: MBVs represent an abundant and potent source of vesicles with strong potential for hard tissue regeneration.

## 26. Isolation and characterisation of breast cancer patient-derived stromal extracellular vesicles

Emily J. Moss, Mahmoud K. Eldahshoury, Jessica L. Haigh, James R. Boyne

Leeds Beckett University, Leeds, UK.

Introduction: Breast cancer (BC) is the most prevalent cancer in the UK. While five-year survival rates are high for localised disease (~90%), outcomes remain poor for metastatic BC (~25%), particularly in aggressive subtypes such as triple-negative BC (TNBC). The tumour microenvironment (TME) plays a key role in BC progression, with cancer-associated fibroblasts (CAFs) promoting tumour growth, chemoresistance, and immunosuppression. Recently, CAF-derived small extracellular vesicles (sEVs) have gained attention for their role in TME modulation via intercellular communication. This study investigated the connection between CAF-derived sEVs and metastasis, with a focus on their molecular cargo involved in signalling.

Methods and results: CAFs were isolated from TNBC patient samples and paired with fibroblasts from adjacent non-tumour tissues (> 5 cm from the tumour). Several sEV isolation methods were compared, including size exclusion chromatography (SEC), ultracentrifugation (UC), and EXODUS - a platform that combines negative pressure oscillation with membrane vibration. Characterisation followed Minimal Information for Studies of Extracellular Vesicles (MISEV) guidelines, and proteomic profiling was conducted using Data-Independent Acquisition Mass Spectrometry (DIA-MS). Both SEC and EXODUS yielded sEVs with higher purity and concentration compared to UC, and provided robust proteomic profiles. Distinct differences in protein cargo were observed between TNBC CAF-derived sEVs and fibroblasts from non-tumour tissue.

Conclusion: In summary, we established a robust sEV isolation pipeline for patient-derived cells and identified unique proteomic signatures in CAF-derived sEVs. Ongoing studies are investigating how these sEVs influence metastatic progression in BC models.

## 27. Intercellular signalling in obesity: adipose-derived EV miRNA communication with skeletal muscle

Joshua Price, Michael Macleod, Thomas Nicholson, Caitlin Ditchfield, Simon Jones

University of Birmingham, Birmingham, UK.

Introduction: We hypothesise that in obesity, adipose-derived extracellular vesicles (ADEVs) drive pathological changes in skeletal muscle, thereby exacerbating sarcopenia. Our objective was to determine whether ADEVs are implicated in sarcopenic-associated muscle changes and to identify the potential cargo responsible.

Methods and results: Adipose-conditioned media (ACM; 24 h) were generated from subcutaneous adipose tissue collected from lean [body mass index (BMI) < 25] or non-lean (BMI > 25) patients with osteoarthritis. ADEVs were isolated from ACM by ultracentrifugation and characterised using nanoparticle tracking analysis and ExoView. Primary myoblasts were differentiated into multinucleated myotubes and cultured for 24 h in complete growth medium containing either vehicle or 1.5 × 10^8^ ADEV/ml derived from lean or non-lean ACM. Myotube RNA was isolated using Trizol and subjected to bulk RNA sequencing (Lexogen). ADEV small RNA was extracted using the mRNeasy Tissue/Cells Advanced Micro Kit (Qiagen) and analysed by small RNA sequencing [Beijing Genomics Institute (BGI)]. Myotube thickness following ADEV treatment was assessed by desmin staining and fluorescent microscopy. ADEV concentration was increased in lean individuals compared to non-lean individuals (*P* < 0.05). Differential expression analysis revealed 201 upregulated and 94 downregulated genes in myotubes treated with non-lean ADEVs *vs.* lean ADEVs. Non-lean ADEVs exhibited an altered miRNA profile, with upregulation of miR-193b-5p, miR-155-5p, and miR-150-5p confirmed by quantitative real-time polymerase chain reaction (qPCR). Furthermore, non-lean ADEVs promoted a reduction in myotube thickness (*P* < 0.05).

Conclusions: Non-lean ADEVs induce detrimental changes in skeletal muscle, leading to both phenotypic changes and associated transcriptomic shifts. The miRNA cargo of ADEVs may be responsible for these changes and is currently under further investigation.

## 28. Extracellular vesicle glycoproteins for early detection of clinically significant prostate cancer

Demi Pritchard^1^, Anastasia Hepburn^2^, Adriana Buskin^2^, Janne Leivo^3^, Haiyan An^1^, Shareen H Doak^1^, Laura E Thomas^1^, Rakesh Heer^4^, Rajan Veeratterapillay^5^, Gokul KandaSwamy^6^, Jason P Webber^1^

^1^Swansea University, Swansea, UK. ^2^Newcastle University, Newcastle, UK. ^3^University of Turku, Turku, Finland. ^4^Imperial College London, London, UK. ^5^Department of Urology Newcastle, Newcastle, UK. ^6^Department of Urology Swansea, Swansea, UK.

Introduction: Prostate cancer (PCa) is the most common cancer to affect men. Accurate diagnosis often requires biopsy to distinguish clinically significant disease from indolent tumours, thereby preventing overtreatment. We previously demonstrated that extracellular vesicle (EV) surface proteoglycans can regulate PCa progression. We therefore hypothesise that an altered EV glycoprofile could serve as a marker to identify patients with clinically significant PCa.

Methods: EVs were isolated by ultracentrifugation from PCa cell lines (LNCaP, Du145, and PC3) and non-cancerous cell lines (PNT2 and BPH1), and characterised in accordance with published guidelines. EV glycoprofiles were assessed using a 95-lectin array and confirmed by plate-based immunoaffinity capture with lectin detection. Additional analyses were performed on EVs secreted frominduced pluripotent stem cell (iPSC)-derived PCa organoid models. Serum samples were collected from patients undergoing diagnostic testing for PCa. EVs were isolated from patient serum using size exclusion chromatography prior to glycoprofile assessment.

Results: Compared with EVs from non-cancerous cells, PCa EVs displayed increased levels of galactose, N-acetylgalactosamine, and N-acetylglucosamine. Similar glycan enrichment was observed in EVs from MYC [myc proto-oncogene (MYC), Basic-Helix-Loop-Helix (BHLH) Transcription Factor]-overexpressing PCa organoids, suggesting a link between altered glycoprofiles and disease progression. Preliminary data further indicated that serum-derived EVs from individuals with clinically significant PCa displayed distinct glycoprofiles compared to those from patients with indolent disease.

Conclusion: An altered EV glycoprofile, associated with MYC overexpression, is linked to PCa progression. EV surface glycans hold promise as biomarkers for the early detection of clinically significant PCa.

## 29. Circulating extracellular vesicles in the pregnant ewe and fetus during late gestation

Natalie Jiraskova^1^, Anna L.K. Cochrane^2^, Rachael C. Crew^2^, Youguo Niu^2^, Abigail L. Fowden^2^, Dino A. Guissani^2^, Priya Samuel^1^, Alison J. Forhead^1^

^1^Oxford Brookes University, Oxford, UK. ^2^University of Cambridge, Cambridge, UK.

Communication between the mother, placenta, and fetus is essential for a healthy pregnancy. Plasma extracellular vesicle (EV) concentrations were 50-fold higher in pregnant compared to non-pregnant women and increased towards term. However, little is known about EVs in the fetal circulation. This project characterises EVs extracted from maternal and fetal plasma near term in a sheep model of pregnancy. Jugular venous plasma from pregnant ewes and umbilical arterial plasma from their fetuses were collected at 130 and 144 days of gestational age (dGA; n = 3 per time point), with term being 145-147 dGA. EVs were isolated by size exclusion chromatography. Immunoblotting and immunogold transmission electron microscopy (TEM) confirmed the presence of EV markers CD63 and CD81 in maternal and fetal plasma. Nanoparticle tracking analysis (NTA) showed that EV concentrations were significantly lower in fetal compared to maternal plasma [mean ± standard deviation (SD): 8.9 ± 2.4 × 10^9^ *vs*. 1.14 ± 0.6 × 10^11^ particles/mL, *P* = 0.002, regardless of dGA]. Fetal plasma EVs were larger in diameter than maternal EVs (140.1 ± 22.1 *vs.* 107.6 ± 8.6 nm, *P* = 0.007). These size differences were confirmed quantitatively by TEM (*P* = 0.002; minimum EVs counted per group = 50). No significant differences in EV concentration or size were observed between 130 and 144 dGA in either maternal or fetal plasma. In conclusion, maternal and fetal plasma EVs differ in concentration and size during late gestation in sheep. The functional significance of these differences in EV profiles remains to be investigated.

## 30. Does the expression of CD63 in extracellular vesicles present in serum differ between thyroid pathologies?

Hannah Beattie^1^, James England^2^, John Greenman^1^, Victoria Green^1^

^1^Centre for Biomedicine, Faculty of Health Sciences, Hull York Medical School, University of Hull, Hull, UK. ^2^Department of Otorhinolaryngology, Head and Neck Surgery, Hull University Teaching Hospitals NHS Trust Hull, Hull, UK.

Introduction: Previous studies using a tissue-on-chip device quantified small extracellular vesicles (sEV) released from thyroid tissues, finding no differences in size or concentration, but differences in sEV content. To determine whether sEV release from tissue is reflected in the serum of the same patients [thyroid cancer (n = 6), Graves’ disease (n = 6), and benign thyroid (n = 6)], we employed the optical particle analysis instrument CX-300 (Paraytec, York, UK).

Method: Serum was diluted 6.25- and 12.5-fold in phosphate buffered saline (PBS). Fifty microlitres of diluted serum was incubated with 2.5 µL fluorescein isothiocyanate (FITC)-conjugated anti-human CD63 antibody (BioLegend) for 60 min. Unbound antibody was removed using a ParaySelect^TM^ column (Paraytec), which selectively retains labelled particles. Eluted particles were analysed using the CX-300 (three 60-second runs per sample).

Results: One sample displayed high baseline fluorescence, preventing analysis. In the remaining samples, differential expression of CD63 was observed between pathologies., Serum from Graves’ disease patients contained significantly more CD63^+^ particles than serum from thyroid cancer patients (*P* < 0.05). Significant differences were also observed across all three patient groups.

Conclusion: The CX-300 fluorescence-based particle analysis system enabled quantification of CD63^+^ particles in serum. CD63 expression varied between thyroid pathologies, in contrast to earlier tissue-on-chip studies that used nanoparticle tracking analysis, which does not assess marker expression. Ongoing work aims to use a two-colour CX-300 system to investigate the expression of an additional tetraspanin marker, CD81.

## 31. Development of melt electrowritten fibrous scaffolds with controlled release of osteocyte-derived extracellular vesicles for bone repair

Mathieu Y. Brunet^#^, Carolina S. Martins^#^, Rawan Almasri, Luke Madden, Mimma Maggio, Lorraine O’Driscoll, David A. Hoey

Trinity College Dublin, Dublin, Republic of Ireland. ^#^Authors contributed equally.

Harnessing additive manufacturing to produce structured scaffolds that guide tissue regeneration has emerged as a promising therapeutic approach for bone repair. In particular, melt-electrowritten (MEW) scaffolds have demonstrated strong regenerative potential when combined with a nanoneedle-hydroxyapatite (nnHA) coating. In parallel, bone cell-derived extracellular vesicles (EVs) have gathered growing attention in recent years as key physiological mediators and promising acellular nanotherapeutics for bone repair. This study investigated the functionalisation of different nnHA-MEW-scaffold microarchitectures with osteocyte-derived EVs to further enhance bone healing. EVs were isolated from the conditioned medium of MLO-Y4 osteocytic cells via ultracentrifugation (110,000 × g, 90 min). Characterisation confirmed the collection of sub-200 nm vesicles by nanoparticle tracking analysis (NTA) and the presence of EV-enriched tetraspanin markers CD9, CD63, and CD81 (flow cytometry). Polycaprolactone (PCL) MEW were then manufactured and coated with nnHA using different microarchitectures (600 µm square pores with 200 µm offset, and 600 µm square pores with no offset) prior to EV functionalisation via collagen I coating. Osteogenic assessment of human mesenchymal stem cells after 21 days, using alizarin red and picrosirius red staining, showed consistent osteogenic potential across both scaffold designs. Comprehensive 2-week release studies were performed to determine the influence of scaffold microarchitecture and EV concentration on release profiles [flow cytometry and confocal imaging following 1,1’-dioctadecyl-3,3,3’,3’-tetramethylindodicarbocyanine (DiD) lipid staining]. A dose-dependent release was observed from day 14 onward, with both architectures showing sustained release. However, scaffolds with the 200 µm offset released significantly more EVs than the no-offset scaffolds from day 14 (*P* < 0.0001). Together, these findings demonstrate the successful development of EV-functionalised MEW scaffolds, offering a novel platform for the controlled delivery of EVs to promote bone defect repair.

## 32. Isolation and characterisation of extracellular vesicles in malaria: implications for pathogenesis and biomarker discovery

Muna Abubaker, Toby Aaron, Joseph Morgan, Sowmya Chinta, Alex Couchman, Berna Sayan, Niroshini Nirmalan, Arijit Mukhopadhyay

University of Salford, Manchester, UK.

Malaria, caused by Plasmodium parasites transmitted through mosquito bites, remains a global health challenge affecting millions worldwide. Despite progress in treatment and prevention, major gaps persist in understanding malaria pathogenesis and identifying reliable diagnostic biomarkers. Extracellular vesicles (EVs), which play critical roles in cell communication and disease progression, have emerged as potential diagnostic tools. This study focused on isolating and characterising small extracellular vesicles (sEVs) from Plasmodium falciparum (K1 strain)-infected red blood cells (iRBCs) and uninfected red blood cells (RBCs) cultured *in vitro*. EV isolation was achieved using a combination of differential centrifugation and size exclusion chromatography (SEC). The resulting sEVs were characterised using nanoparticle tracking analysis (NTA), transmission electron microscopy (TEM), bicinchoninic acid (BCA) protein assays, and western blotting. Our findings revealed a clear correlation between parasitaemia levels and sEVs release. As parasitaemia increased, iRBCs secreted significantly more sEVs, demonstrating a strong positive relationship between infection severity and vesicle release. In contrast, uninfected RBCs released substantially fewer sEVs, with over 70% fewer vesicles compared to iRBCs (*P* = 3.8 × 10^-5^). Regardless of the parasite stage (ring, trophozoite, or schizont), more than 75% of sEVs were under 200 nm in size. Preliminary western blotting also detected Plasmodium falciparum aldolase and Flotillin-1 in iRBC-derived sEVs, though further validation is required. These findings suggest that sEVs could serve as accessible biomarkers for early malaria detection, evaluation of disease severity, monitoring of treatment responses, and the development of new disease management strategies.

## 33. Müller glia-derived EVs promote recovery in retinal ganglion-like cells derived from hESC retinal organoids *in vitro*

Karen Eastlake^1,2,3^, William Lamb^1,2,3^, Hari Jayaram^1^, Astrid G. Limb^1^

^1^National Institute for Health Research (NIHR), Biomedical Research Centre Moorfields Eye Hospital NHS Foundation Trust and UCL Institute of Ophthalmology, London, UK. ^2^Department of Global Alliances and Collaboration, Global Ophthalmology Research. ^3^Development, Santen Incorporated, Emeryville, CA, USA.

Introduction: We have recently demonstrated that membrane-bound extracellular vesicles (EVs) released by Müller glia contain microRNAs (miRNAs) and proteins that may regulate anti-apoptotic processes and provide neuroprotection in retinal degenerative conditions. To evaluate the therapeutic potential of Müller glia-derived EVs, we investigated their neuroprotective effects in retinal ganglion-like cells isolated from human embryonic stem cell (hESC)-derived retinal organoids *in vitro*.

Methods: Retinal ganglion-like cells were isolated from hESC-derived retinal organoids and characterised using immunofluorescence staining for retinal ganglion cell (RGC) markers [class III β-tubulin (βIII-tubulin), bromine azide (Brn3), γ-synuclein, RNA-binding protein with multiple splicing (RBPMS), Thy-1 cell surface antigen (Thy1), Paired Box 6 (PAX6)]. Cytotoxicity was induced with Nmethyl-D-aspartic acid (NMDA), followed by treatment with Müller cell-derived EVs. Neurite features, including number and length, were assessed using ImageJ.

Results: The isolated RGC-like cells expressed typical RGC markers, including βIII-tubulin, Brn3b, RBPMS, γ-synuclein, Thy1, and NMDAR1. NMDA exposure significantly reduced average neurite length in these cells. Treatment with Müller cell-derived EVs resulted in a significant increase in average neurite length compared to NMDA-only controls.

Conclusions: RGC-like cells derived from hESC retinal organoids provide an effective *in vitro* model for studying RGC degeneration. EVs released by Müller glia promote the survival and neurite outgrowth of RGC-like cells. Further work is needed to identify the mechanisms by which Müller glia-derived EVs exert their therapeutic effects. These findings support the potential of EV-related therapies for RGC damage in retinal degenerative conditions such as glaucoma.

## 34. Impact of drug-to-lipid ratio on microfluidic production of DOX·HCL encapsulating liposomes

Toby M. Hyden-Shepherd, Goran T. Vladisavljevic, Owen G. Davies

Loughborough University, Loughborough, UK.

Introduction: Extracellular vesicles (EVs) are promising candidates for next-generation drug delivery vehicles (DDVs). However, their clinical application is currently limited by inconsistent drug loading and insufficient quantification of exogenous cargo. To address these challenges, we tested a microfluidic method for co-loading the chemotherapeutic drug doxorubicin (DOX·HCL) during liposome production. Liposomes were selected due to their rapid production and scalable, reproducible manufacturing.

Methods: A 3D-printed microfluidic chip was applied to produce populations of large unilamellar vesicles (LUVs) composed of zwitterionic phospholipid 1,2-Dioleoyl-sn-glycero-3-phosphocholine (DOPC). LUVs were produced by microfluidic mixing of an aqueous phase (DI water) and an organic phase (5 µg/mL DOPC in 99.9% ethanol) at a total flow rate of 12 mL/min. Drug-to-lipid ratios were varied by adjusting flow rate ratios (FRR) from 0.15 to 0.3. Particle size and concentration were analysed by nanoparticle tracking analysis (NTA), while DOX·HCL encapsulation efficiency (EE%) was determined by absorbance at 480 nm.

Results: Increasing the FRR from 0.15 to 0.3 significantly improved EE% from 0.20% (±0.14) to 4.64% (±0.03) (*P* < 0.001). Increasing FRR also correlated with a significant decrease in particle size from 119.7 (±4.41) to 101.77 (±6.93) (*P* < 0.05). In contrast, particle concentration significantly decreased from 5.3 × 10^12^ (±7.87 × 10^11^) at FRR 0.15 to 5.3 × 10^11^ (±1.31 × 10^11^) at FRR 0.30 (*P* < 0.01).

Conclusions: We demonstrated the successful encapsulation of DOX·HCl in LUVs using a microfluidic platform. Future work will aim to further increase encapsulation efficiency by incorporating anionic phospholipids into the lipid formulation and exploring the fusion of DOX·HCl-loaded liposomes with EVs to generate a novel nanocarrier drug delivery system (DDS).

## 35. Hijacking breast cancer cell-derived extracellular vesicles for enhanced therapeutic delivery of curcumin

Malika Singh^1^, Ellie Hitchcock^1^, Rebecca Lees^2^, Owen G. Davies^1^

^1^Loughborough University, Loughborough, UK. ^2^NanoFCM Co. Ltd, Nottingham, UK.

Curcumin, a natural polyphenol, possesses anti-inflammatory and anti-oncogenic characteristics. However, its poor solubility limits clinical application. Extracellular vesicles (EVs) are a promising drug delivery system to improve curcumin’s pharmacokinetics and therapeutic efficacy. This study sought to evaluate the loading efficiency and functional activity of curcumin encapsulated in breast cancer-derived EVs (BC-EVs).

EVs were isolated using ultracentrifugation (UC) from human breast cancer cell lines MDA-MB-231 (triple-negative) and T47D (luminal-A). EVs were characterised using nanoparticle tracking analysis, nano-flow cytometry (NanoFCM), and western blotting. Passive loading was performed by incubating EVs with curcumin (1 mg/mL) for 30 min at room temperature, followed by UC (120,000 × g) to remove unbound curcumin. Curcumin association with EVs was quantified spectrophotometrically (420 nm). Solubility and stability of EV-curcumin (1.1 × 10^11^ particles/mL) in Dulbecco’s phosphate-buffered saline (DPBS) over 150 min were compared with curcumin-only controls. RT-PCR analysis of p53 and p21 expression was conducted to provide a preliminary assessment of therapeutic efficacy.

MDA-MB-231- and T47D-derived EVs differed significantly in particle yield (1.71 × 10^10^ *vs.* 3.89 × 10^11^) and mean size (76.18 nm *vs.* 80.32 nm). Marker expression also varied: CD9 (6.9% *vs.* 3.1%) and CD63 (13.0% *vs.* 6.4%). Maximum curcumin loading efficiency was higher in MDA-MB-231 EVs (41.19%) compared with T47D EVs (30.77%). The two EV populations enhanced curcumin solubility four-fold and five-fold and improved stability by 1.6- and 1.8-fold, respectively, relative to free curcumin. EV-curcumin (100 µg/mL) significantly downregulated p53 and p21 expression in MDA-MB-231 (*P* < 0.0001) and T47D (*P* < 0.01) cells.

Overall, our findings demonstrate that BC-EVs markedly improve the solubility and stability of curcumin. Despite source-dependent differences in EV yield, size, and tetraspanin expression, both EV types showed therapeutic potential.

## 36. The Parkinson’s disease-associated protein DJ-1 regulates EV function under oxidative stress by modulating their abundance and protein cargo

Thomas Page^1^, Saskia Bakker^2^, Ivana Milic^1^, Andrew Devitt^1^, Mariaelena Repici^1^

^1^School of Biosciences, College of Health & Life Sciences, Aston University, Aston Triangle, Birmingham, UK. ^2^Advanced Bioimaging RTP facility, University of Warwick, Coventry, UK.

DJ-1 mutations cause familial Parkinson’s disease (PD). While DJ-1 is involved in protecting cells against oxidative stress, its precise role in PD pathogenesis remains unclear. Elevated DJ-1 levels have recently been detected in extracellular vesicles (EVs) isolated from biological fluids of PD patients, providing a link between DJ-1, EVs, and disease progression. To investigate this, we examined the effects of DJ-1 knockout (KO) on EV populations derived from neuronally differentiated SH-SY5Y cells under basal and rotenone-induced oxidative stress. EV abundance was assessed by flow cytometry, protein content by mass spectrometry, and functional activity by macrophage migration assays. Results showed that small EV numbers increased in both wild-type (WT) and DJ-1 KO cells following rotenone exposure, though with distinct dose-dependent patterns. In WT cells, small EV release increased 2.25-fold at 5 nM and 2.36-fold at 10nM rotenone compared with controls. In DJ-1 KO cells, increases occurred at higher doses: 3.48-fold at 10 nM and 2.19-fold at 25 nM rotenone. Proteomic analysis at 10 nM rotenone revealed 117 proteins differentially expressed between genotypes. Mitochondrial health was compromised in DJ-1 KO cells compared with WT, without a corresponding increase in cell death, suggesting that altered EV responses contribute to stress adaptation. Furthermore, DJ-1 KO modified the ability of EVs to stimulate macrophage migration, reversing the effect observed with WT EVs. These findings identify DJ-1 as a regulator of EV biogenesis and/or uptake under oxidative stress. They provide new insight into the early mechanisms of DJ-1-linked PD, highlighting a previously unrecognised role for DJ-1 in EV-mediated intercellular communication.

## 37. Aberrant extracellular vesicle signalling by endothelial cells - a potential pathological mechanism in cerebral small vessel disease (SVD)?

Rebecca L. Robertson^1^, Ronja Kremer^1^, Dani Ruiz Gabarre^1^, Garyfallia Gouna^1^, Anna Adamska^1^, Bethany Geary^2^, Lorraine Work^3^, Joanna Wardlaw^1^, Anna Williams^1^

^1^University of Edinburgh, Edinburgh, UK. ^2^University of Dundee, Dundee, UK. ^3^University of Glasgow, Glasgow, UK.

Cerebral small vessel disease (SVD) is common and increases the risk of cognitive impairment and stroke, but its pathological mechanisms remain poorly understood. In SVD, endothelial cells lining the brain’s small vessels become dysfunctional, influencing not only adjacent but also more distant oligodendrocytes. This disruption alters oligodendrocyte function and contributes to the white matter changes seen on MRI in humans. These observations suggest that impaired endothelial cells could secrete factors, potentially via altered extracellular vesicle (EV) release, to communicate with oligodendrocytes.

This project aims to determine whether, and how, EVs are involved in SVD and how they affect oligodendrocytes. EVs were isolated from circulating blood plasma to identify changes in protein cargo between Atp11bKO and wild-type rats using size exclusion chromatography and proteomics. In 2-month-old rats, no differences were observed in EV size, but the proteomic signatures differed between genotypes. Ongoing work is examining signals from dysfunctional endothelial cells during disease progression by analysing EVs from 6-month-old rats. In addition, EVs isolated from cultured primary endothelial cells were significantly smaller in Atp11bKO rats compared with wild-type controls. Planned experiments will assess the effects of these EVs, or their component proteins, on cultured oligodendrocytes to determine their influence on downstream cellular function. Investigating the role of endothelial EVs in SVD is important, as the molecular mechanisms underlying this pathology are still poorly defined. A clearer understanding of these processes may ultimately inform the development of effective therapies.

## 38. The impact of hypoxia on macrophage-derived extracellular vesicles in placental implantation studies

Richard Odusanya, Andrew Devitt, Stephane Gross

Aston Institute for Membrane Excellence, School of Biosciences, Aston University, Birmingham, UK.

Introduction: Placental development begins with the attachment of the blastocyst, followed by trophoblast invasion into the maternal endometrium. Successful implantation is essential for foetal development. Communication between the developing embryo and the mother, including immune regulation, plays a critical role in this process. Macrophages influence the maternal-foetal environment partly through the release of extracellular vesicles (EVs), which are lipid-bound particles carrying proteins, lipids, and RNAs essential for intercellular communication. Our work seeks to characterise macrophage-derived EVs (MØdEVs).

Methods and results: We first sought to establish the conditions that promote macrophage EV release and to examine their molecular cargo, with the goal of understanding their role in regulating the maternal-foetal environment. This study investigated the effects of oxygen availability and macrophage origin on MØdEVs *in vitro*. Macrophages differentiated from THP-1 and MonoMac-6 monocytes were cultured under 1%, 8%, and 21% oxygen. EVs were then isolated and characterised. The results show that hypoxia increases MØdEV release and that macrophages derived from MonoMac-6 cells produce more EVs than those derived from THP-1 cells.

Conclusions: These findings identify hypoxic stress as a key regulator of EV release from macrophages, with potential implications for immune responses and placental implantation under low-oxygen conditions. Future studies will examine MØdEVs from polarised macrophages (M1 and M2) under hypoxia, assessing their composition and impact on trophoblast migration and implantation. Such work could yield important insights into managing pre-eclampsia and improving pregnancy outcomes.

## 39. Investigating the role of EVs in wound repair: developing corneal and dermal wound healing models

Sasha Kieran^1^, Abigail Adams^1^, James Wolffsohn^2^, Andrew Devitt^1^

^1^Aston Institute for Membrane Excellence, School of Biosciences, Aston University, Birmingham, UK. ^2^College of Health & Life Sciences Aston University, Birmingham, UK.

Introduction: With ageing, the body’s ability to repair tissue damage and control inflammation declines, increasing the risk of chronic wounds. Extracellular vesicles (EVs) have been shown to promote wound healing and resolve inflammation, though the mechanisms remain poorly understood. This study aims to define the role of EVs and their cargo in wound healing, with a particular focus on ocular and dermal models.

Methods and results: Using a scratch assay, apoptotic cell-derived secretome (comprising EVs and soluble factors) significantly improved wound healing in HeLa cell monolayers. Building on this, a modified scratch assay was developed to measure wound closure in immortalised corneal epithelial and dermal fibroblast cells, providing a more physiologically relevant wound healing model. The effects of osmolarity changes - mimicking dry eye conditions - on wound repair were assessed, as well as the combined effects of increased osmolarity and ultraviolet (UV) exposure. Complete closure was observed in both corneal and dermal models by 48 h, whereas combined osmolarity changes and UV exposure significantly impaired wound repair. Future work will focus on generating EVs from immune cell lines (T cells and monocytes) and stem cells to further investigate their role in wound healing.

Conclusions: We established and optimised a cellular wound healing model to compare corneal and dermal repair under normal conditions and in the presence of external stressors that hinder repair. Further investigation is necessary to understand how EVs and their cargo influence these processes, with the potential to inform new strategies for enhancing wound healing in clinical settings.

## 40. Active extracellular vesicles - exploring the power of EV enzymes in boosting wound healing and macrophage migration

Abigail Elizabeth Adams, Ivana Milic, Andrew Devitt

Aston Institute for Membrane Excellence, School of Biosciences, Aston University, Birmingham, UK.

Introduction: Wound healing is a complex, tightly regulated process, and disruptions can lead to chronic wounds that severely impact quality of life. Extracellular vesicles (EVs) are increasingly recognised for their role in mediating communication within the wound environment, but the exact mechanisms remain unclear. Recent studies suggest that 5/12/15-lipoxygenase (LOX) enzymes carried within EV cargo may promote macrophage migration, thereby influencing wound healing. This study investigates the role of EVs and their LOX cargo in macrophage migration and wound closure.

Methods and results: *In vitro* scratch assays showed that apoptotic cell-derived secretome (containing EVs and soluble factors) significantly promoted wound closure in HeLa cell monolayers. After 60 h, wounds treated with the secretome closed by 40%, compared to only 5% in controls. Transwell assays with HL60 monocyte-derived macrophages showed enhanced migration towards the secretome, which was markedly reduced by inhibition of 5/12/15-LOX. Pre-treatment of the secretome with a LOX inhibitor reduced macrophage migration by 80%. Likewise, macrophages pre-treated with the inhibitor demonstrated significantly impaired migration towards the apoptotic secretome.

Conclusion: This study highlights the role of apoptotic secretome and LOX enzymes in promoting wound closure, likely through macrophage migration and debridement. The presence of 5/12/15-LOX within EV cargo suggests a potential therapeutic target for chronic wound treatment. Further studies are needed to explore the interaction between EV-associated LOX enzymes and pharmacological LOX inhibition to enhance our understanding of EV-mediated wound repair.

## 41. Obese adipose-derived extracellular vesicles drive skeletal muscle atrophy: implications for obesity and age-related muscle loss

Michael Macleod, Caitlin Ditchfield, Tom Nicholson, Josh Price, Simon W. Jones

University of Birmingham, Birmingham, UK.

Sarcopenic obesity, characterised by excess adiposity together with diminished skeletal muscle mass and function, is associated with frailty and chronic inflammation. Previous work from our group implicated dysregulated crosstalk between adipose tissue (AT) and skeletal muscle (SkM) as a driver of sarcopenic obesity, and increasing evidence demonstrates that extracellular vesicles (EVs) mediate intercellular communication. Here, we aimed to characterise AT-EV profiles in lean and obese subjects, relate these profiles to measures of adiposity and inflammation, explore their effects on SkM, and identify underlying mechanisms of action.

Adipose-conditioned media (ACM) were prepared from *ex vivo* tissue explants, and EVs were isolated by ultracentrifugation. EV profiles were analysed using ExoView and nanoparticle tracking analysis. Human primary SkM cells were treated with ACM or AT-EVs for 24 h (post-differentiation) or 8 days (during differentiation). Myotube thickness and nuclear fusion were measured as functional readouts. RNA was extracted from treated myotubes to assess gene expression.

Obese AT released fewer EVs than lean AT, although visceral and fat pad depots consistently yielded more EVs than subcutaneous AT, supported by trends in EV-associated protein concentrations. ExoView surface marker analysis revealed depot-specific differences in AT-EV profiles. Compared with untreated controls, obese AT-EVs significantly reduced myotube thickness and upregulated atrophic and inflammatory gene expression, suggesting modulation of catabolic and anabolic pathways relevant to SkM hypertrophy. Further interrogation of these pathways in the contexts of adiposity, exercise-mimetic stimulation, and age-related muscle loss will advance mechanistic understanding of sarcopenic obesity.

Obese, obese AT-EVs induce atrophic signatures in SkM, highlighting the need for further research into EV-mediated crosstalk in sarcopenic obesity.

## 42. Proteomic analysis of *ex vivo* glioblastoma tumour-derived extracellular vesicles

Shaun Hanley^1^, Ourania Gkanatsiou^1^, Kesley Attridge^2^, Andrew Devitt^1^, Ivana Milic^1^

^1^Aston University, Birmingham, UK. ^2^University of Birmingham, Birmingham, UK.

Introduction: Glioblastoma (GBM) is the most common and aggressive brain tumour, with a poor prognosis: average survival is ~15 months, and the 5-year survival rate is < 10%. One major factor underlying this poor outcome is the ability of GBM to create an immunosuppressive tumour microenvironment (TME) through several mechanisms, including secretion of GBM-derived extracellular vesicles (GBM-EVs). Additionally, GBM cell line models have limited heterogeneity of patient tumours and therefore do not fully capture the complexity of *in vivo* disease. Therefore, we aimed to investigate the GBM-EV proteome from *ex vivo* GBM tumours to identify possible immunomodulatory proteins contributing to TME immunosuppression.

Methods and results: Transport medium was collected from 58 surgically excised GBM tumours, and GBM-EVs were isolated using size exclusion chromatography. Molecular subtyping classified tumours into four clinical subtypes (3 mesenchymal, 16 classical, 13 pro-neural, 15 with overlapping classical and pro-neural features, and 11 unclassified). Proteins were separated by gel electrophoresis and analysed by liquid chromatography-mass spectrometry. Protein identification was performed with Progenesis QI for proteomics (v.4.1.6675.48614) and functional annotation using FunRich (v3.1.3). Across all samples, 1,397 unique proteins were identified, 54 of which appear to be related to immune function.

Conclusions: Further analysis will reveal the commonalities and differences in GBM-EV proteomes across GBM subtypes and identify proteins that may regulate immunomodulation within the TME. This represents the largest proteomic study of *ex vivo* GBM-EV to date and may identify novel treatment targets for GBM.

## 43. Investigating the role of S100P in placental extracellular vesicle release and maternal immunomodulation

Natasha Louise Baker^1^, Andrew Devitt^2^, Stephane Gross^1^

^1^School of Biosciences, Aston University, Birmingham, UK. ^2^Aston Institute for Membrane Excellence, School of Biosciences, Aston University, Birmingham, UK.

Introduction: The S100 protein family contains 30 isoforms of low-molecular-weight (9-14 kDa) calcium-binding proteins. Among them, S100P has been implicated in cell migration, invasion, and immunosuppression within the tumour microenvironment. We hypothesise that S100P may regulate similar functions during placental development, as it is well documented that S100P is expressed in trophoblasts - the cells responsible for establishing the placenta. We therefore investigated the potential role of S100P in trophoblast extracellular vesicle (EV) production under different environmental conditions, as well as the functional implications of S100P carriage in these vesicles.

Methods and results: Clones of the HTR-8 trophoblast cell line stably expressing exogenous S100P were cultured alongside non-transfected controls. EVs were analysed in the conditioned medium and following ultracentrifugation. Vesicle abundance was estimated using Bodipy-FL maleimide staining and flow cytometry. Preliminary results showed that S100P expression correlates with at least a threefold increase in EV release.

Conclusions: As S100P is shown to interact with the actin-myosin cytoskeleton, our findings indicate that its upregulation may trigger outward membrane blebbing, thereby enhancing vesicle release. Future work will determine whether S100P is packaged within trophoblast-derived EVs and assess its biological role, specifically whether S100P-containing vesicles modulate the maternal-foetal immune interface in healthy and pathological pregnancies.

## 44. Active extracellular vesicles: towards therapeutic use in wound healing & inflammation

Samuel Charles Hulme^1^, Ritu Raj^1^, Ivana Milic^2^, Andrew Devitt^1,2^

^1^Aston University, Birmingham, UK. ^2^Aston Institute for Membrane Excellence, Aston University, Birmingham, UK.

Introduction: Extracellular vesicles (EVs) are small membrane-bound structures released by cells to mediate intercellular communication. They transport a diverse range of cargo, including cytokines, nucleic acids, and enzymes such as lipoxygenases (LOX). Lipoxygenases play key roles in regulating biological processes, including wound healing and inflammation. Our study investigates the therapeutic potential of EVs carrying lipoxygenases (e.g., 5-LOX, 12-LOX, and 15-LOX) in wound healing and inflammation.

Methods: LOX plasmids for HEK293 transfection were generated through DH5-α E.coli transformation (ampicillin-resistant), followed by plasmid purification and restriction enzyme digestion. Commercially purchased HEK293 adherent cells were transfected with LOX or green fluorescent protein (GFP)-based plasmid DNA using Metafectene/Lipofectamine reagents. LOX protein expression was confirmed in transfected cells by Western blotting.

Results: Western blot analysis demonstrated high expression of LOX proteins in transfected HEK293 cells. Further investigation is required to confirm LOX expression in stably transfected cells and assess their incorporation into secreted EVs. Fluorescence microscopy further revealed that HEK293 cells are suitable for both transient and stable GFP transfection, with Lipofectamine proving more efficient than Metafectene.

Conclusion: Lipoxygenases play an important role in inflammation and wound healing, and EVs may serve as a promising delivery system for LOX-based therapies. Western Blotting and fluorescence imaging confirmed successful expression of LOX and GFP proteins in HEK293 cells, providing a strong foundation for generating therapeutic EVs using similar approaches.

## 45. Determining the effects of electroporation parameters on extracellular vesicle properties

Malika Singh, Ghazaleh Mazaheri-Tehrani, Ignacio Martin-Fabiani, Mark Lewis, Owen G. Davies

Loughborough University, Loughborough, UK.

Introduction: Extracellular vesicles (EVs) hold great potential as drug delivery systems. Electroporation is a widely applied method for active EV-drug loading. However, current protocols often lack standardisation and their effects on EV integrity remain unclear. This study aimed to investigate the impact of electroporation parameters (voltage, pulse number, and pulse width) on EV characteristics to inform the development of an effective loading protocol.

Methods: EVs were isolated from C2C12 murine myoblasts via ultracentrifugation, and recovery was validated according to Minimal Information for Studies of Extracellular Vesicles (MISEV) guidelines. EVs were suspended in Neon Transfection Buffer (TB) and electroporated (EP) under varying conditions: voltage (V: 500, 750, 1,000 V), pulse number (PN: 1, 2, 3 pulses), and pulse width (PW: 10, 20, 30 ms). EVs were subsequently washed in Dulbecco’s phosphate-buffered saline (DPBS) by ultracentrifugation (UC) to assess whether native profiles could be restored. Following electroporation, protein concentration, zeta potential (ZP), particle concentration, and size were measured.

Results: Significant changes in EV size, concentration, and zeta potential were observed following suspension in TB. Native EV profiles were not restored following DPBS washing; in general, EV size increased and ZP became less negative following application of V, PN and PW. TB-suspended samples by UC resulted in an 89% loss of protein content, whereas electroporation did not significantly affect protein levels.

Conclusions: Our results suggest that EV profiles are adversely affected by the transfection process, with changes not reversed by washing.

## 46. Characterisation of paediatric thymic Tregs and their extracellular vesicles with potential for immunosuppressive therapy in autoimmune disorders

Anaïs Makos^1^, Jordan Bazoer^2^, Henry Barrett^1^, Rafael Guerrero^3^, Dan Hawcutt^3^, Lesley Smyth^2^, Oksana Kehoe^1^

^1^Centre for Regenerative Medicine Research, School of Medicine, Keele University at the RJAH Orthopaedic Hospital, Oswestry, UK. ^2^School of Medicine and Biosciences, University of West London, London, UK. ^3^The Alder Hey Children’s NHS Foundation Trust, Liverpool, UK.

Introduction: Regulatory T cells (Tregs) are crucial for maintaining immune balance and offer promising therapeutic options for autoimmune diseases due to their suppressive capacities. Extracellular vesicles (EVs) derived from Tregs may offer a practical alternative, offering improved stability and ease of delivery. This study aimed to isolate and expand Tregs from paediatric thymuses, extract their EVs, and evaluate their immunosuppressive potential.

Methods and results: Tregs were isolated and expanded from paediatric thymuses (n = 5), with purity confirmed by flow cytometry markers (CD4, CD8, CD25, and CD127) and further characterised using CXCR3, TIGIT, and CTLA4. Growth analysis from the first thymus sample showed successful expansion under four conditions: complete media with/without human serum (HS) and with/without rapamycin. Tregs proliferated without HS, and purity remained comparable with and without rapamycin. EVs were isolated from expanded Tregs and characterised by cryo-transmission electron microscopy (cryo-TEM), nanoparticle tracking analysis (NTA), and tetraspanin markers. Functional assays demonstrated that both thymic Tregs and their EVs can suppress T effector cell (Teff) proliferation.

Conclusion: Our study successfully isolated and characterised Tregs and their EVs from paediatric thymuses, showing their potential for immunosuppressive therapy. Tregs and their EVs could be valuable tools for treating autoimmune diseases such as rheumatoid arthritis. EV-based therapies offer practical advantages over cell-based approaches, highlighting their potential as an innovative strategy for immune modulation.

## 47. Characterising the proteomes of *Salmonella* outer membrane vesicles under intestinal conditions

Alastair Scott^1,2^, Mark Stevens^1,2^, Garry Blakely^1,2^, Prerna Vohra^1,2^

^1^The Roslin Institute and Royal (Dick) School of Veterinary Studies, University of Edinburgh, Easter Bush, Edinburgh, UK. ^2^Institute for Immunology and Infection Research, School of Biological Sciences, Charlotte Auerbach Road, University of Edinburgh, Edinburgh EH9 3FF, UK.

*Salmonella* enterica is a bacterial pathogen of global importance. Different serovars of S. enterica cause diseases ranging from acute enteritis (e.g., S. Typhimurium in humans, cattle, and pigs) to systemic infections (e.g., S. Dublin in cattle and S. Choleraesuis in pigs, occasionally affecting humans). Similar to other Gram-negative bacteria, *Salmonella* produces outer membrane vesicles (OMVs), which contribute to pathogenesis by fusing with host cells to deliver virulence factors that facilitate infection and manipulate the immune response. Protein packaging into OMVs can be influenced by environmental conditions, including those present in the gut. Here, we characterised changes in the OMV proteomes of S. Typhimurium, S. Dublin, and S. Choleraesuis strains of defined virulence, comparing cultures grown in standard laboratory conditions (lysogeny broth) with those exposed to intestinal-level bile (1%) from cattle and pigs. Our goal was to identify serovar-specific differences that may relate to the type of disease caused. OMVs were purified from late exponential-phase cultures using density gradient ultracentrifugation and quantified by nanoparticle tracking analysis. Periplasmic proteins were also isolated at the same growth phase using cold osmotic shock. Data-independent acquisition mass spectrometry revealed both serovar- and condition-specific differences in OMV proteomes. Across all serovars, exposure to bile resulted in additional periplasmic proteins being packaged into OMVs, along with an increased presence of cytoplasmic proteins. These findings suggest a reduction in selective periplasmic packaging and potential protein leakage across the inner membrane. Furthermore, 40 proteins were considerably more abundant under bile conditions. Ongoing investigations are exploring how these alterations in OMV protein composition may affect virulence.

## 48. Measuring surface markers on activated platelet EVs using novel fibre-optic surface plasmon resonance technology

Neline Kriek^1^, Ryan Pink^2^, Jonathan Gibbins^1^

^1^University of Reading, Reading, UK. ^2^Oxford Brookes University, Oxford, UK.

Introduction: The abundance, surface markers, and messenger molecules of extracellular vesicles (EVs) in blood may reflect the health and activation status of the tissues from which they originate. Platelet-derived EVs play roles in blood homeostasis and are implicated in conditions such as cardiovascular disease and cancer. Fibre-Optic Surface Plasmon Resonance (FO-SPR; FOx BioSystems) is a relatively new technology where antibodies are immobilised on a fibre-optic sensor to capture proteins or EVs. Binding events, including subsequent antibody interactions, are measured in real-time as changes in reflected light. In this study, we performed dynamic analyses of EVs from resting and activated platelets, examining differences in their abundance and surface markers.

Methods and results: EV release from platelets increased following stimulation with strong activators such as TRAP-6. We analysed EVs from untreated (resting) and TRAP-6-activated Platelet-Rich Plasma (PRP) samples using the WhiteFOx system, employing the generic EV marker CD9 and platelet-specific markers CD41 and CD61 (subunits of integrin αIIbβ3), as well as CD62P (P-selectin, exposed on activated platelets) to capture and/or detect platelet-derived EVs. Activation increased both the total number of EVs and the proportion of platelet-derived EVs, accompanied by elevated signals for CD41, CD61, and CD62P. These outcomes were validated by flow cytometry.

Conclusions: FO-SPR technology enables profiling of EV surface markers from resting and activated platelets using small plasma volumes, offering a promising approach for the analysis of clinical samples.

## 49. Maternal obesity programs hearts to release miR-15b-5p in extracellular vesicles after ischemia-reperfusion

Adriana Córdova-Casanova, Lucas C. Pantaleão, Isabella Inzani, Youguo Niu, Dino A. Giussani, Denise S. Fernandez-Twinn, Susan E. Ozanne

University of Cambridge, Cambridge, UK.

Introduction: Environmental and nutritional imbalances during fetal and early postnatal life increase the risk of cardiometabolic disease in adult offspring. Although several mechanisms have been proposed, growing evidence highlights the role of miRNAs and extracellular vesicles (EVs) as key mediators. Here, we investigated the interaction between miRNA 15b-5p and EVs in the developmental programming of cardiac metabolism by maternal obesity.

Methods and results: Using a murine model, we studied the effects of maternal obesity during pregnancy on offspring cardiac expression of miR-15b-5p. A Langendorff preparation was used to determine whether miR15b-5p is released in EVs from isolated hearts in response to ischemia-reperfusion (IR) in mice. Uptake of cardiac EVs by cultured cells was assessed using high-content microscopy. Hearts of adult mice born to obese dams exhibited increased levels of miR-15b-5. We further demonstrated that cardiac tissue releases miR-15b-5p during IR, with a proportion of this miRNA packaged within EVs. Release of miR-15b-5p in EVs was higher in adult mice exposed to maternal obesity. Cardiac-like myoblasts (H9c2) and umbilical endothelial cells (HUVECs), but not preadipocyte (3T3-L1), readily internalised DiR-labelled cardiac EVs in a time-dependent manner.

Conclusions: Our findings suggest that miR-15-5p, programmed by maternal obesity, is released during IR and packaged into EVs. These EVs are preferentially taken up by cardiac and vascular cells, suggesting possible autocrine/paracrine communication between ischaemic hearts and the peripheral vasculature.

## 50. Cyclodextrin’s role in modifying cancer cell extracellular vesicles

Júlia Kvasznicza^1^, Afrodité Németh^1^, Gréta Lilla Bányai^1^, Levente Szőcs^2^, Tamás Garay^1^

^1^Pázmány Péter Catholic University, Budapest, Hungary. ^2^Cyclolab Cyclodextrin Research and Development Laboratory Ltd, Budapest, Hungary.

Introduction: Cyclodextrins (CDs) have a unique toroidal structure that enables them to encapsulate lipids and hydrophobic molecules. Through their interactions with membrane lipids, CDs may modulate extracellular vesicle (EV) production and composition, thereby influencing intercellular communication and potentially affecting cancer progression.

Methods: The sensitivity of WM983A and WM983B melanoma cells, and human epidermal keratinocytes (HaCaT) and human embryonic kidney 293 (HEK-293) non-tumourigenic cells to RAMEB (randomly methylated β-cyclodextrin) was assessed using the sulphorhodamine B (SRB) viability assay in Dulbecco’s modified Eagle’s medium (DMEM) supplemented with either standard fetal bovine serum (FBS) or EV-depleted FBS. EVs from WM983A and WM983B cells were generated under control and RAMEB-treated conditions, isolated by ultracentrifugation, and characterised using nanoparticle tracking analysis (NTA), Qubit protein assay, and sulpho-phospho-vanillin (SPV) assay. HaCaT and Mel Pt-4 pre melanoma cells (previously shown to produce functional EVs) were then treated with EVs derived from HaCaT, Mel Pt-4 pre control, and RAMEB-treated Mel Pt-4 pre cells.

Results: HaCaT and HEK-293 cells showed no sensitivity to RAMEB. WM983A and WM983B cells remained viable with RAMEB in standard FBS but exhibited reduced viability in EV-depleted FBS. RAMEB-treated WM983A and WM983B cells produced EVs with reduced lipid content, while particle count, size, and protein content remained unchanged. EVs generated under different conditions had no significant impact on cell migration.

Conclusion: RAMEB selectively modulates EV lipid content without compromising cell viability, suggesting its potential as a tool to influence EV composition. This property may be clinically useful for altering EV-mediated communication in cancer and other diseases, providing a novel approach to modulating EV profiles without impairing cell health.

## 51. Engineering the next generation of biologically active vascular grafts

Justine Clarke^1^, Mara Boumpouli^2^, Manuel Salmeron-Sanchez^1^, Niall Paterson^2^, Catherine Berry^1^

^1^University of Glasgow, Glasgow, UK. ^2^Terumo Aortic, Renfrew, UK.

Introduction: Cardiovascular diseases (CVDs) are the leading cause of death globally. Although vascular grafts have shown great promise, they remain susceptible to thrombosis over time. In collaboration with Terumo Aortic, this study explores the potential of mesenchymal stromal cell (MSC)-derived extracellular vesicles (EVs) as a biologically active coating for their Polyethylene Terephthalate (PET) endovascular grafts used in aneurysm treatment.

Methods and results: MSCs were cultured under normoxic (21% O_2_) and hypoxic (1% and 5% O_2_) conditions to generate EVs enriched in angiogenic factors. The EVs were isolated and characterised by size, concentration, and cytokine cargo. While EV size was comparable across all conditions, 5% hypoxia yielded a higher concentration of EVs. Angiogenic potential was assessed using human umbilical vein endothelial cells (HUVECs) cultured on Matrigel with media supplemented with either normoxic or hypoxic EVs. Tube formation analysis showed that 5% hypoxic EVs promoted angiogenesis (positive control), whereas normoxic EVs performed similarly to the negative control.

To assess immobilisation, graft samples were coated with a proprietary formulation enabling EV binding via integrins. EVs were fluorescently stained with carboxyfluorescein diacetate succinimidyl ester (CFSE) (green) and imaged using confocal microscopy. MSC-EVs successfully adhered to the graft surface, with the woven topography influencing their spatial distribution.

Conclusion: Hypoxic conditions enhanced the production of EVs with angiogenic activity, which could be immobilised onto PET graft material. Future work will further assess their therapeutic potential by (i) analysing EV metabolites, proteomics, and miRNA cargo, and (ii) evaluating the interactions of HUVECs and peripheral blood mononuclear cells (PBMCs) with immobilised MSC-EVs on graft material.

## 52. Advancing extracellular vesicle analysis: microfluidic approaches for cancer diagnostics

Gréta Lilla Bányai^1^, András Szabó^1^, Anikó Gaál^2^, Csaba Pongor^1^, András Merényi^3^, Tamás Garay^1,4^

^1^Pázmány Péter Catholic University, Faculty of Information Technology and Bionics, Budapest, Hungary. ^2^Research Centre for Natural Sciences, Institute of Materials and Environmental Chemistry, Biological Nano-chemistry Research Group, Budapest, Hungary. ^3^Department of Emergency Medicine, Semmelweis University, Budapest, Hungary. ^4^Department of Internal Medicine and Oncology, Division of Oncology, Semmelweis University, Budapest, Hungary.

Introduction: Extracellular vesicles (EVs) mirror the molecular features of their parent cells and can be readily obtained from body fluids, making them a valuable non-invasive diagnostic source. Their characteristics change under pathological conditions, positioning them as promising prognostic and predictive biomarkers. However, plasma-derived EVs originate from diverse cell types, requiring highly sensitive detection methods. With the growing demand for robust, cost-effective diagnostic tools, microfluidic devices have emerged as an attractive option for monitoring cancer-related EV profile changes.

Methods: Plasma samples from patients with pancreatic, prostate, and colorectal cancers were processed using size exclusion chromatography (SEC) to isolate EV-rich fractions. Nanoparticle tracking analysis (NTA) was performed to measure mean particle size, size distribution, and concentration, while protein and lipid content were quantified across patient groups. For microfluidic analysis, EVs were stained with a protein-specific dye, followed by removal of unbound dye through a second SEC round. Fluorescence microscopy captured signal distribution along the channel diameter, and fluorescence intensity data were fitted to an asymmetric sigmoid curve. From these profiles, the amplitude and growth rate of the averaged function were calculated.

Results: NTA did not effectively distinguish between patient groups, nor did protein or lipid quantification provide meaningful diagnostic differentiation. In contrast, microfluidic profiling of EVs yielded distinct signatures for each cancer type.

Conclusions: Microfluidic techniques enable the characterisation of EVs across a broader range of properties than conventional methods, positioning them as promising, robust, and cost-effective tools for EV-based diagnostics. This approach holds potential for future application in biomarker-driven cancer diagnostics.

## 53. A characterised immortalised mesenchymal stromal cell (MSC) line to facilitate standardisation of MSC-EV immunomodulatory potency assays

Krishalle Richmond^1,2^, Melanie Moore^2^, Yuan Zhao^2^, Murat Eravci^2^, Min Fang^2^, Lesley Smyth^3^, Samir Shawgy Ayoub^3^, Anna Nowocin^1^

^1^MHRA, London, UK. ^2^University of East London, London, UK. ^3^University of West London, London, UK.

Introduction: Bioactive components secreted by mesenchymal stromal cells (MSCs), including extracellular vesicles (MSC-EVs), are recognised as key mediators of MSC immunomodulatory and regenerative functions. However, progress in understanding the complex mechanisms of MSC-EVs’ action is impeded by their inherent heterogeneity, which arises from variations in EV bioprocessing factors such as cell source. Moreover, the proliferative capacity, therapeutic potency, and EV secretion ability of primary MSCs decline over their finite lifespan. To overcome these limitations, we developed an immortalised MSC line (ImmMSC) that ensures a continuous supply of MSC-EVs while minimising production variability.

Methods and results: The ImmMSC line was established by transducing human bone marrow-derived MSCs (BMMSCs) with a lentiviral vector encoding the telomerase gene (hTERT). The immunomodulatory activity, growth characteristics, and phenotype of ImmMSCs and their EVs were characterised in comparison with primary BMMSCs. ImmMSCs exhibited the expected plastic adherence and stable surface marker identity. The line also demonstrated trilineage differentiation into chondrocytes, osteocytes, and adipocytes following 2 weeks of differentiation. Importantly, nanoparticle tracking analysis and functional assays revealed that ImmMSCs outperformed BMMSCs in EV production and batch-to-batch consistency. ImmMSCs also remained responsive to tumor necrosis factor α (TNFα) licensing, which led to higher expression of all major tetraspanins in secreted EVs. The line has been extensively passaged and has retained these characteristics throughout expansion.

Conclusion: This ImmMSC line provides a platform for standardising MSC-EV bioprocessing and enables detailed investigations into the immunomodulatory and regenerative functions of MSC-EVs, as well as the production of reference reagents for bioactivity.

## 54. Identification of EV biomarkers of senescence and development of sensors to detect them

Benjamin Raven, Caroline Evans, Graham Leggett, Daniel W. Lambert

University of Sheffield, Sheffield, UK.

Cellular senescence contributes to the development of age-related diseases. Senolytic and senomorphic drugs are being developed to target senescent cells and their secretions. Effective clinical application, however, requires sensitive methods to detect and quantify senescence. The aim of this project is to identify novel extracellular vesicle (EV) biomarkers of senescence and develop new sensor technologies for their detection. MRC5 (Medical Research Council strain no. 5) fibroblasts were exposed to ionising radiation, and senescence acquisition was analysed using senescence-associated size exclusion chromatography (SEC) assessment. EVs were isolated from senescent and proliferating cells using SEC and ultracentrifugation. These were characterised by nano-flow cytometry (nFCM), atomic force microscopy (AFM), and nanoparticle tracking analysis (NTA). Proteomic analysis of MRC5 cells and their EVs was conducted to identify biomarkers of senescence. Quartz Crystal Microbalance (QCM) analysis was also performed on EVs from senescent and proliferating cells to investigate the potential of QCM for EV biomarker discovery. We confirmed that senescence can be induced in MRC5 cells using ionising radiation and that sufficient EVs can be obtained for proteomic analysis. However, EV yields were relatively low, requiring further concentration for liquid chromatography-mass spectrometry (LC-MS) compared to other EV analysis methods. QCM yielded promising results, indicating that EVs can be captured on an antibody-coated silica disc, providing a platform for developing sensitive sensors for EV biomarkers. EVs thus represent a promising source of non-invasive biomarkers for senescence detection, supporting the clinical translation of senolytic and senomorphic therapies. Moving forward, we aim to use proteomic analysis to identify novel EV biomarkers of senescence and integrate these into next-generation sensor technologies for non-invasive senescence quantification.

## 55. How do tumour-derived extracellular vesicles interact with the developing nervous system and alter pain processing in cancer survivors?

Hannah K Jackson^1,2^, Anna Grabowska^3^, Victoria James^4^, Federico Dajas-Bailador^1,2^, Beth Coyle^3^, Gareth Hathway^1,2^

^1^Pain Centre Versus Arthritis, University of Nottingham, Medical School, Queen’s Medical Centre, Nottingham, UK. ^2^School of Life Sciences, Medical School, Queen’s Medical Centre, Nottingham, UK. ^3^School of Medicine, Biodiscovery Institute, University of Nottingham, Nottingham, UK. ^4^School of Veterinary Medicine and Science, University of Nottingham, Nottingham, UK.

Introduction: Childhood cancer-related pain and its treatment remain insufficiently addressed, largely due to a limited understanding of the underlying mechanisms. Although chemotherapy-induced peripheral neuropathic pain (CIPN) has been studied in adults, its impact during early development is poorly understood. Growing evidence suggests that tumour-derived factors may influence pain processing. This study investigates how tumour-derived extracellular vesicles (EVs) interact with the developing nervous system and modulate pain processing in childhood brain tumour survivors.

Methods: Dose-response curves and IC50 values were determined for several medulloblastoma (MB) cell lines using standard-of-care chemotherapeutic agents: vincristine, etoposide, cisplatin, and lomustine. EVs were isolated via size exclusion chromatography and characterised by western blotting, direct stochastic optical reconstruction microscopy (dSTORM), and electron microscopy. We then examined how chemotherapy-treated MB EVs influenced axon development and function in primary embryonic day 16.5 dorsal root ganglion (DRG) neurons *in vitro*.

Results: While EVs from treated and untreated cells had similar size and morphology, chemotherapy at low concentrations significantly increased EV secretion in MB cell lines. Moreover, co-culturing DRG neurons with these EVs induced neuronal death and elevated calcium excitation in DRGs.

Conclusions: Standard chemotherapeutic agents significantly increased EV release from MB cells, and these EVs promoted DRG neuronal death and enhanced calcium excitation. Future studies will investigate the impact of these EVs on pain responses and neural maturation in healthy animals, focusing on EV biodistribution and chemotherapy-induced changes in EV cargo within the nervous system.

## 56. Exploring stable sEV miRNA biomarkers for prenatal alcohol exposure using human neural cell models

Toby Aarons, Aadya Lakhani, Amalia Kachramani, Pramitha Sivaprakash, Berna Sayan, Arijit Mukhopadhyay

University of Salford, Manchester, UK.

Introduction: Prenatal alcohol exposure (PAE) is a global health concern that adversely affects fetal brain development and increases the risk of fetal alcohol spectrum disorder (FASD). Reliable biomarkers for PAE could enable early detection and intervention, improving management of long-term developmental challenges. Small extracellular vesicles (sEVs), particularly sEV-derived miRNAs, offer a promising source for minimally invasive biomarker discovery, as they may reflect enduring brain changes even after ethanol clearance. This study explores sEV miRNA responses to ethanol exposure using human neural cell models of PAE.

Methods: SH-SY5Y cells were differentiated into mature neurons, chronically exposed to ethanol (100 mM), and subsequently cultured without ethanol to evaluate persistent sEV miRNA alterations. sEVs were isolated using size exclusion chromatography and characterised by fluorescence nanoparticle tracking analysis (f-NTA) and western blotting. Real-time quantitative polymerase chain reaction (qPCR) was used to analyse miRNA from both cells and sEVs.

Results: Differentiated SH-SY5Y cells, expressing NeuN, MAP2, and β3-tubulin, demonstrated transcriptional shifts in miRNA expression after 7 days of ethanol exposure, with some alterations persisting 7 days post-ethanol. sEVs, collected at multiple stages of differentiation, were primarily < 200 nm and CD81/CD63-positive. Dysregulated miRNAs (miR-9-5p, miR-134-5p, miR-485-5p) were identified in sEVs following ethanol treatment.

Conclusions: Detecting stable, PAE-specific molecular alterations after ethanol exposure is vital for biomarker development. Our findings reveal a panel of sEV miRNAs as promising and stable biomarkers for PAE, which are currently being validated in more complex neural models.

## 57. Exploring the role of extracellular vesicles in Alzheimer’s disease pathogenesis using Down syndrome *in vitro* models

Ana Muñiz-García, Aoife Murray, Dean Nizetic

Queen Mary University of London, London, UK.

Introduction: Alzheimer’s disease (AD) is one of the most common causes of dementia, yet no effective treatment currently exists. Due to the dose-sensitive role of the amyloid precursor protein (APP) gene, located on chromosome 21 (Chr21), people with Down Syndrome (DS) face an increased risk of developing early-onset AD (EOAD). Other genes on Chr21, such as Beta-Secretase 2 (BACE2), may further modulate this risk. Extracellular vesicles (EVs) have been strongly implicated in AD pathology; however, their specific role remains unclear. Using our well-established DS *in vitro* models, we started investigating the role of EVs in AD pathogenesis.

Methods and results: We employed a previously validated human *in vitro* model of AD pathogenesis (Alic *et al.* 2021). Cerebral organoids (COs) were generated from induced pluripotent stem cells (iPSCs) derived from euploid controls (D21), DS (T21), or DS with only two BACE2 copies (T21Δ7), following the methodology described by Lancaster *et al.* (2013). Organoids were cultured for 90 days. Small EVs (sEVs) were isolated from CO lysates, conditioned media, or frozen foetal brain tissues (D21/T21, 18-19 weeks gestation) by differential ultracentrifugation. Characterisation was performed using nanoparticle tracking analysis, nano-flow cytometry, and western blotting. sEVs carried both endo-lysosomal and mitochondrial proteins. T21 sEVs showed elevated levels of Chr21-encoded proteins. Notably, T21 CO-derevied EVs contained increased levels of an Aβ epitope-containing protein (6E10), with the effect further enhanced by BACE2 mutation. No significant differences in this epitope were detected between D21 and T21 foetal brain samples.

Conclusions: These preliminary results demonstrate that DS *in vitro* models provide a valuable platform for investigating the role of EVs in AD pathogenesis.

## 58. Modulation of extracellular vesicle release from endothelial cells under inflammatory stimulation

Laura Sture, Chong Yun Pang, Neil Sheerin, Simi Ali

Newcastle University, Newcastle, UK.

Introduction: Extracellular vesicles (EVs) are increasingly recognised as key contributors to both the progression and resolution of disease. The endothelium plays a critical role in inflammation, yet the functions of endothelial cell-derived EVs during inflammatory responses remain poorly understood. This study aimed to investigate the characteristics of EVs released by endothelial cells under inflammatory stimulation.

Methods and results: The endothelial cell line HMEC-1 (human microvascular endothelial cell line) was stimulated with 20 ng/mL interferon-γ (IFN-γ), and EVs were isolated from conditioned culture media (CCM) using size exclusion chromatography. EVs were characterised by nanoparticle tracking analysis (NTA), transmission electron microscopy and western blotting, confirming the presence of 50-250 nm particles positive for canonical EV markers CD9, CD63, and TSG101. NTA showed that EV release decreased by 70% post IFN-γ stimulation compared to untreated cells, after normalisation to cellular protein at the time of CCM collection (*P* < 0.001). Surface marker profiling using the Miltenyi MACSPlex kit showed increased expression of human leukocyte antigen (HLA) class II molecules on EVs from IFN-γ-treated cells (*P* < 0.001), with mass spectrometry identifying HLA-DR and HLA-DP specifically. Further proteomic and miRNA analyses of EV cargo are ongoing. Functional assays are being conducted to evaluate the effects of these EVs on endothelial proliferation and wound healing.

Conclusion: EVs released from endothelial cells under inflammatory conditions exhibit a distinct profile compared to those from untreated cells. The presence of HLA class II molecules on EVs derived from IFN-γ-treated cells suggests a potential for modifying immune responses. These findings support the hypothesis that EVs can exert either anti- and pro-inflammatory effects depending on their cellular origin and microenvironment.

## 59. The role of EV-associated miRNAs in medulloblastoma recurrence

Sree V. Dokka^1^, Hannah K. Jackson^2,3^, Connor T. Levers^2^, Beth Coyle^2^

^1^University of Nottingham, Nottingham, UK. ^2^Children’s Brain Tumour Research Centre, Biodiscovery Institute, University of Nottingham, Nottingham, UK. ^3^School of Life Sciences, University of Nottingham, Nottingham, UK.

Introduction: Recurrent medulloblastoma (MB) is particularly prevalent in the aggressive Group 3 and 4 subgroups, where the five-year survival rate remains below 10%. Emerging evidence suggests that extracellular vesicles (EVs) carrying molecular cargo such as microRNAs may drive MB progression and recurrence by activating oncogenic pathways and promoting tumour cell proliferation.

Methods and results: Next-Generation Sequencing (NGS) of cerebrospinal fluid (CSF) samples from primary and recurrent MB identified four differentially enriched miRNAs. Differential expression analysis with DESeq2 revealed seven candidate miRNAs, including miR-187-3p and miR-483-3p, which showed significant differential expression in recurrent samples compared to primary samples. Analysis of EVs derived from primary and recurrent Group 3 (D425, D458) and Group 4 (CHLA-01, CHLA-01R) cell lines showed that only miR-187-3p was consistently abundant in both patient CSF and cell line EVs. Quantitative polymerase chain reaction (PCR) further confirmed elevated miR-187-3p expression in recurrent cell lines relative to their primary counterparts. Target prediction identified BARX2, a transcription factor implicated in cell adhesion, as a putative target of miR-187-3p. Gene expression and survival analyses using R2 Genomics showed that high BARX2 expression in Groups 3 and 4 MB correlated with poorer overall survival. Importantly, CSF analysis confirmed BARX2 as the only consistently upregulated target across all patient subgroups.

Conclusion: These findings suggest that EV-associated miR-187-3p may function as an oncomiR through regulation of BARX2. These results provide a foundation for functional studies to validate the targeting of BARX2 by miR-187-3p and to explore how EV-associated miR-187-3p influences MB cell behaviour, supporting its potential as a therapeutic target.

## 60. Exploring probiotic-derived extracellular vesicles as novel therapeutic agents in the management of inflammatory bowel disease

Robert Matthews^1^, Nouhad El OUazzani^2^, Samir Ayoub^2^, David Guilliano^3^, Nati Garrido Mesa^4^, Lesley Ann Smyth^1^

^1^School of Medicine and Biosciences, University of West London, London, UK. ^2^School of Health, Sport and Bioscience, University of East London, London, UK. ^3^School of Life Sciences, University of Westminster, London, UK. ^4^School of Life Sciences, Pharmacy and Chemistry, Kingston University, Kingston Upon Thames, UK.

Introduction: Inflammatory Bowel Disease (IBD) is an umbrella term encompassing Crohn’s Disease (CD) and Ulcerative Colitis (UC), both of which are characterised by chronic inflammation of the gastrointestinal (GI) tract. Probiotics have been shown to improve IBD patient outcomes, with the yeast Saccharomyces boulardii (S. boulardii) being one of the most extensively studied in clinical settings. S. boulardii exerts beneficial effects through multiple mechanisms, including the immunomodulation of host enterocytes and local immune cells within the GI tract. Despite these favourable outcomes, limitations exist, particularly in immunocompromised IBD patients, where S. boulardii use has been linked to fungemia. Additionally, live probiotic administration is associated with high variability in dosing. These challenges highlight the need for a safer alternative to the whole yeast, and yeast-derived extracellular vesicles (EVs) may offer such a solution.

Methods and results: We demonstrated that S. boulardii constitutively produces ~150 nm EVs expressing HSP70. EVs were isolated from yeast cultures using Ultracentrifugation (100,000 × g) or Ultrafiltration (100 kDa), followed by Size Exclusion Chromatography (SEC, 70 nm). EV size and protein content were characterised by Nanoparticle Tracking Analysis (NTA) and Western blotting, respectively. *In vitro* assays showed that S. boulardii EVs exhibited no immunogenicity when tested on murine dendritic cells and the human Acute Monocytic Leukaemia cell line THP-1. Notably, S. boulardii EVs suppressed lipopolysaccharides (LPS)-induced IL-6 production while promoting IL-10 production, consistent with an anti-inflammatory effect.

Conclusions: S. boulardii-derived EVs are non-immunogenic and possess inherent anti-inflammatory properties, indicating their potential as novel therapeutic agents for IBD management.

## 61. Messenger and message: exploring the role of extracellular vesicles in inter-organismal communication during arbuscular mycorrhizal symbiosis in *Medicago*

Serena Qiao, Sam Holland, Alice Boussaroque, Zongyu Gao, Mary Woodward, Ronelle Roth

University of Oxford, Oxford, UK.

Introduction: Extracellular vesicles (EVs) are increasingly recognised as conserved mediators of cross-kingdom communication, such as during plant-pathogen interactions. Enclosed by a lipid bilayer, these nanovesicles transport diverse molecular cargoes to the extracellular space, where they are taken up by and modulate the function of recipient cells. In plant-pathogen interactions, plants release EVs containing defence-related cargoes such as messenger RNA (mRNA), stress-related proteins, and small RNAs, which target and attenuate microbial virulence. However, the role of EVs in mutualistic associations - such as those between arbuscular mycorrhizal (AM) fungi and most land plants - remains less well understood. Evidence supporting EV involvement in beneficial plant-fungal interactions comes from transmission electron microscopy and tomography studies, which have shown that multivesicular body (MVB)-derived exosomes and other EV-like particles are released into the shared extracellular matrix between fungal arbuscules and the host-derived peri-arbuscular membrane (PAM).

Methods: EVs were purified from apoplastic wash fluids of AM fungal-colonised and mock-inoculated roots, followed by LC-MS/MS and RNAseq analyses of purified EVs.

Results and conclusions: Using liquid chromatography coupled to tandem mass spectrometry (LC-MS/MS) combined with spatiotemporal analysis of EV biomarkers and associated small RNAs, we provide evidence for a dynamically regulated release of EVs and their sRNA cargo throughout the lifespan of the arbuscule. Moreover, analysis of host sRNA target genes suggests EV-contained sRNAs play a role in modulating fungal lipid utilisation during symbiosis.

## 62. Surface glycans of small extracellular vesicles *in vitro* and *in vivo* increase in early- and late-stage colon cancer, providing novel biomarkers for potential blood biopsy

Jamie Cooper, Bethy Airstone, Emanuela Carollo, Susan Ann Brooks, Ryan Pink

Oxford Brookes University, Oxford, UK.

Introduction: Colon cancer is the fourth most commonly diagnosed cancer, accounting for 11% of all cases. The release and role of plasma small extracellular vesicles (sEVs) in cancer are well documented. Here, we show that colon epithelial cancer cells and their sEVs have surface glycans that increase with cancer stage, mirroring patterns observed in plasma samples from colon cancer patients.

Methods: Colon cancer cell lines were probed for surface glycans using lectins and analysed by confocal microscopy and flow cytometry. Nano-flow cytometry and single-particle interferometry were employed to detect these glycans on EVs. Total plasma EVs from healthy individuals, stage I-II non-metastatic patients, and stage IV metastatic colon cancer patients were assessed for lectin binding by nano-flow cytometry.

Results: Glycan binding was increased in cells and sEVs from metastatic colon cancer lines compared to non-metastatic and immortalised normal colon cells, correlating lectin binding with metastatic potential. In patient-derived plasma sEVs, lectin binding was significantly higher in colon cancer patients than in healthy controls, highlighting its potential as a biomarker for blood-based cancer detection.

Conclusion: This research suggests that sEV surface glycans have potential diagnostic utility as early biomarkers of colon cancer, particularly through the detection of lectin-positive sEVs.

## 63. Functional effects of cows’ milk-derived particles on human inflammatory responses *in vitro*

Amy Fenton^1^, Dory Kovacs^1^, Aprill Park^1^, Robert C. Fowkes^2^, Ryan C. Pink^3^, Charlotte Lawson^1,4^

^1^Comparative Biomedical Sciences, Royal Veterinary College, London, UK. ^2^Department of Small Animal Clinical Sciences, Michigan State University, East Lansing, MI, USA. ^3^School of Biological and Medical Sciences, Oxford Brookes University, Oxford, UK. ^4^School of Pharmacy and Biomedical Sciences, University of Central Lancashire, Preston, UK.

Introduction: Chronic inflammation is a hallmark of cardiovascular and obesity-related diseases and is a major cause of morbidity and mortality in developed countries. While milk and dairy consumption have previously been implicated in the obesity crisis, more recent research suggests that dairy products may confer health benefits. We have previously shown that, in addition to milk fat globules (MFGs), raw cows’ milk also contains extracellular vesicles (EVs).

Method and results: Milk was collected from individual cows at different stages of lactation (measured as Days in Milk, DIM). EVs in whole milk were quantified by flow cytometry and then isolated by sequential ultracentrifugation. The isolated EVs were applied to THP-1 monocytes or HMEC-1 (human microvascular endothelial cell line) endothelial cells, and EV uptake, reactive oxygen species (ROS) production, and cytokine release were assessed. RNA was extracted from the EVs, and miRNA sequencing was performed.

DIM positively correlated with total and annexin-V+ EVs, while DIM negatively correlated with ROS production stimulated by milk-derived EVs. A correlation was observed between EV exposure and ROS production, but not cytokine release. miRNA sequencing of EVs isolated from cows at early, mid, and late lactation revealed distinct differences in miRNA cargo across lactation stages, which may explain the functional variation observed.

Conclusion: These findings suggest that the molecular cargo of milk-derived particles should be further examined to better understand the potential effects of dairy consumption on vascular health in chronic inflammatory conditions.

## 64. Isolation of T cell-derived extracellular vesicles from patients with rheumatoid arthritis

Henry Barrett^1^, Lesley Smyth^2^, Anaїs Makos^3^, Charlotte Hulme^3^, Oksana Kehoe^3^

^1^Keele University, Robert Jones and Agnes Hunt Orthopaedic Hospital, Keele, UK. ^2^University of East London, London, UK. ^3^Keele University, Robert Jones and Agnes Hunt Orthopaedic Hospital, Keele, UK.

Introduction: Regulatory T cells (Tregs) are CD4+CD25+ T helper cells that play a central role in maintaining self-tolerance, and their dysfunction contributes to autoimmune disorders such as rheumatoid arthritis (RA). A deeper understanding of Treg biology in RA may improve diagnostics and treatment. Tregs can promote immune tolerance through the release of extracellular vesicles (EVs) - small, lipid-bound structures capable of transmitting suppressive signals in the absence of direct Treg contact. This highlights their potential as a novel cell-free therapeutic approach.

Methods and results: CD4+CD25+CD127- Tregs were isolated from the peripheral blood of healthy donors and RA patients, then expanded in TexMACS medium supplemented with rapamycin (100 nM), IL-2 (1,000 IU/mL), gentamycin (50 μL/mL), and anti-CD3/CD28 Dynabeads. Flow cytometry confirmed no difference in starting purity between healthy (N = 4) and RA (N = 4) samples, and CD4+CD25+CD127- purity was maintained after 24-30 days of expansion. At isolation, Tregs from healthy (N = 3) and RA (N = 3) donors showed no differences in PD-1, OX40, or CTLA-4 expression. However, PD-1 positivity in RA samples decreased by 12% after expansion. Treg-derived EVs were isolated from conditioned media via ultracentrifugation and ultrafiltration. Conditioned media were obtained by resuspending Tregs in ultracentrifuged expansion medium and culturing them on anti-CD3/CD28-coated plates for 24 h.

Conclusion: We successfully isolated and expanded Tregs from both healthy and RA donors and generated pre-characterised Treg-EV samples. The next phase of this project will involve confirming Treg-EVidentity according to Minimal Information for Studies of Extracellular Vesicles (MISEV) guidelines and comparing the functionality and molecular composition of Treg-EVs from healthy versus RA donors.

## 65. Does the secretion of sEVs containing differential microRNA levels by medulloblastoma during disease progression suppress neuroinflammation?

Connor T. Levers^1^, Hannah K. Jackson^1^, Ian D. Kerr^2^, Federico Dajas-Bailador^2^, Beth Coyle^1^

^1^Children’s Brain Tumour Research Centre, School of Medicine, University of Nottingham Biodiscovery Institute, Nottingham, UK. ^2^School of Life Sciences, University of Nottingham, Nottingham, UK.

Introduction: Small Extracellular Vesicles (sEVs) are key mediators of intercellular communication and play a central role in establishing a favourable Tumour Microenvironment (TME), including interactions with microglia, the resident macrophages of the Central Nervous System (CNS). Medulloblastoma (MB), the most common malignant paediatric CNS tumour, is stratified into four molecular subgroups defined by distinct genetic drivers and metastatic potential.

Methods and results: Differential expression of miRNAs from sEVs derived from primary (CHLA-01-MED), metastatic (CHLA-01R-MED) MB cell lines and non-malignant foetal neuronal cells (FB83) were analysed via DESeq2. A total of 115 miRNAs were significantly enriched, and 189 were significantly depleted with disease progression. Of particular interest was the consistent depletion of miR-9-5p with increasing malignancy: a 2.8-fold reduction was observed in CHLA-01-MED versus FB83, and a 5.3-fold reduction in CHLA-01R-MED versus CHLA-01-MED. Ranking by normalised counts showed miR-9-5p among the top 5% of miRNAs in FB83 sEVs, top 10% in CHLA-01-MED sEVs, and top 20% in CHLA-01R-MED sEVs. Literature evidence suggests neuronal enrichment of miR-9-5p in sEVs promotes pro-inflammatory polarisation of microglia. We therefore hypothesised that miR-9-5p depletion in MB-derived sEVs could suppress this polarisation, contributing to an immunosuppressive TME. Preliminary co-culture experiments with HMC-3 microglia and CHLA-01R-MED sEVs did not enhance proliferation, in contrast to prior findings where metastatic MB sEVs promoted proliferation and invasiveness in primary and non-malignant cell lines. This suggests MB-derived sEVs exert cell-specific effects.

Conclusion: Future work will investigate cytokine secretion profiles associated with pro- and anti-inflammatory polarisation of HMC-3 cells, as well as morphological changes following co-culture with CHLA-01R-MED sEVs.

## 66. Bone marrow stromal cell-derived extracellular vesicles for bone regeneration

Evangelia Bochti^1^, Monica P. Tsimbouri^1^, Biswajoy Bagchi^2^, Xin Li^2^, Matthew J. Dalby^1^, Catherine Berry^1^

^1^University of Glasgow, Glasgow, UK. ^2^Sphere Fluidics Limited, Cambridge, UK.

Introduction: Bone is the second most transplanted tissue worldwide, with over 4 million procedures performed annually. Bone marrow-derived stromal cells (BMSCs) are widely used in bone tissue engineering (BTE) due to their osteoblastic differentiation potential. Their beneficial effects are partly mediated by paracrine signalling, including extracellular vesicles (EVs). In collaboration with Sphere Fluidics Limited, this project aims to create a novel BMSC-EV microenvironment by encapsulating osteogenically primed EVs within hydrogel beads for cell-free bone regeneration and repair therapies.

Methods and results: BMSCs were osteogenically primed for 28 days using two approaches: (1) chemical induction with osteogenic media, and (2) mechanical induction via nanoscale vibrational displacement. Osteogenic differentiation was verified via protein analysis (Western blotting) and histological staining (alizarin red). BMSC-derived EVs were isolated and characterised by size and concentration. EV cargo is currently being evaluated at cytokine, metabolomic, proteomic, and RNA levels. Osteogenic EVs are also being co-cultured with naïve BMSCs to assess their osteogenic potency. Both chemical and mechanical priming successfully induced osteogenesis in BMSCs. EV isolation was validated, with consistent particle sizes (60-250nm). Differences in cytokine profiles have already been observed between the two induction approaches, with further analyses ongoing at metabolomic, proteomic and RNA levels. Both types of osteogenic EVs are under evaluation for their ability to promote osteogenesis in naïve BMSCs.

Conclusion: BMSC priming successfully induced osteogenesis. Current efforts focus on characterising BMSC-derived EV cargo to identify differences between osteogenic and control conditions, as well as between chemical and mechanical induction methods.

## 67. Investigating extracellular vesicle heterogeneity during arbuscular mycorrhizal symbiosis in *Medicago* truncatula

Alice Boussaroque, Samuel Holland, Ronelle Roth

University of Oxford, Oxford, UK.

Introduction: Extracellular vesicles (EVs) are membrane-bound nanoparticles produced by all kingdoms of life, including plants and fungi. They transport a wide range of bioactive molecules - including proteins, DNA, and RNA - that contribute to cross-kingdom communication. Arbuscular mycorrhizal (AM) symbiosis is an ancient, mutually beneficial relationship between most land plants and fungi of the Glomeromycotina. The presence of EVs at the symbiotic plant-fungal interface suggests a role for EVs in modulating this interaction.

Methods and results: EVs were isolated from AM fungal-colonised roots (Myc) and mock-inoculated roots (Mock) using differential ultracentrifugation. Transmission electron microscopy (TEM) and Nanoparticle Tracking Analysis (NTA) revealed an enrichment of larger EVs in Myc compared to Mock. Two-step ultracentrifugation further separated EVs by size: larger vesicles were recovered in the P40 fraction, whereas smaller vesicles were enriched in the P120-P40 fraction. Both P40 and P120-P40 fractions from Myc contained larger vesicles compared to Mock, suggesting that AM symbiosis induces the production of highly heterogeneous EVs originating from the plant and/or fungus. Plant EVs are commonly classified into exosomes labelled by TETRASPANIN 8 (MtTET8), the plant ortholog of CD63, whereas larger EVs could be labelled by PENETRATION 1/ SYNTAXIN OF PLANT 132 (MtSYP132). Western blotting revealed differential accumulation of these biomarkers in the P40 versus P120-P40 fractions.

Conclusion: The isolation of EV populations with distinct biomarkers, sizes, cargo contents, and biogenetic pathways will facilitate a deeper understanding of EV heterogeneity in AM symbiosis and provide new mechanistic insights into EV-mediated cross-kingdom communication.

## 68. Isolation and characterisation of human amniotic epithelial cell extracellular vesicles for therapeutic repair of human donor hearts and lungs

Nicholas Chilvers^1^, Angel Zhang^2^, Simi Ali^2^, Andrew Fisher^1^, John Dark^2^

^1^Newcastle University / Institute of Transplantation, The Freeman Hospital, Newcastle, UK. ^2^Newcastle University, Newcastle, UK.

Introduction: Transplantation remains the gold-standard treatment for patients with end-stage heart or lung disease. However, over 80% of donor organs are declined due to their susceptibility to ischaemia-reperfusion injury (IRI). Mesenchymal stem cells have demonstrated efficacy in models of cardiac ischaemia and lung disease, with benefits increasingly attributed to paracrine effects mediated by extracellular vesicles (EVs). EVs carry cargo that can suppress inflammatory and apoptotic pathways. Animal studies have shown that EV administration reduces IRI and improves organ function. Human amniotic epithelial cells (hAECs), derived from placentas otherwise discarded, are immunomodulatory and represent a promising EV source.

Methods and results: From donated placentas (n = 31), an average of 123.9 × 10^6^ hAECs were isolated following dissection and digestion. EVs were obtained from day 4 culture media of 50 × 10^6^ thawed or fresh hAECs using ultracentrifugation or size-exclusion chromatography, yielding 1.05 × 10^10^ (n = 5) and 1.62 ×10^10^ particles (n = 5), respectively. Flow cytometry confirmed EV expression of tetraspanins as well as hAEC-specific markers CD29 and SSEA4. Uptake into endothelial cell lines was confirmed using flow and confocal microscopy.

Conclusion and future work: hAEC-derived EVs can be efficiently isolated from human placentas. In future studies, EV doses will be administered during warm reperfusion of human hearts and lungs that are declined for clinical transplantation. The experimental setup enables sophisticated assessment of organ function. Tissue biopsies and perfusate samples will be studied to determine the mechanisms involved. hAEC-EVs hold the promise for optimising marginal donor organs not currently used in transplantation, thereby improving organ availability and outcomes.

## 69. Synovial fluid extracellular vesicles modulate the transcriptome of human articular chondrocytes in osteoarthritis

Caitlin Ditchfield, Joshua Price, Simon Jones

University of Birmingham, Birmingham, UK.

Introduction: Osteoarthritis (OA) is characterised by articular cartilage degradation and joint pain. Extracellular vesicles (EVs) may mediate critical crosstalk between joint tissues in OA.

Methods: OA patients undergoing joint replacement surgery were recruited. Patient-reported pain and quality of life (QoL) were assessed using Oxford Knee Score (OKS) and EQ-5D questionnaires, and patients were categorised into “high pain” or “low pain” groups. Cartilage tissue and synovial fluid were collected, and articular chondrocytes were isolated by collagenase digestion. EVs were isolated from synovial fluid (SF) and characterised by Nanoparticle Tracking Analysis (NTA) and ExoView. Chondrocytes were treated for 24 h with SF-EVs from either “high pain” or “low pain” patients. RNA was subjected to bulk sequencing, and differential expression was analysed using DESeq2. Transcriptional changes were evaluated with Ingenuity Pathway Analysis (IPA), and selected genes of interest were validated by polymerase chain reaction (PCR) or by Luminex analysis of conditioned media.

Results: Lower quality of life was associated with a reduced concentration and increased mean size of SF-EVs (NTA). Tetraspanin levels were elevated on SF-EVs from patients experiencing high pain compared to those reporting low pain (ExoView). Forty-seven genes were differentially expressed between high-pain EV and low-pain EV-treated chondrocytes (logFC < 1.5; *P* > 0.05), including several OA-related genes. IPA highlighted IL4/IL13 signalling, CDK5 signalling, and L1CAM signalling as the most dysregulated pathways, indicating that SF-EVs mediate inflammatory and neuronal signalling.

Conclusions: SF-EV size, concentration, and tetraspanin content vary according to self-reported pain levels. SF-EVs induce differential transcriptomic effects in articular chondrocytes depending on pain severity, influencing inflammatory, angiogenic, and neuronal signalling pathways.

## DECLARATIONS

### Copyright

© The Author(s) 2025.

